# Tunable Spin Qubit Pairs in Quantum Dot–Molecule Conjugates

**DOI:** 10.1021/acsnano.5c00288

**Published:** 2025-03-19

**Authors:** Autumn Y. Lee, Mandefro Teferi, Frida S. Hernandez, Amisha Jain, Tiffany Tran, Kefu Wang, Tomoyasu Mani, Adam M. Schwartzberg, Ming Lee Tang, Jens Niklas, Oleg G. Poluektov, Jacob H. Olshansky

**Affiliations:** 1 Department of Chemistry, Amherst College, Amherst, Massachusetts 01002, United States; 2 Chemical Sciences and Engineering Division, Argonne National Laboratory, Lemont, Illinois 60439, United States; 3 Department of Chemistry, University of Utah, Salt Lake City, Utah 84112, United States; 4 The Molecular Foundry, Lawrence Berkeley National Laboratory, Berkeley, California 94720, United States; 5 Department of Chemistry, University of Connecticut, Storrs, Connecticut 06269-3060, United States

**Keywords:** quantum dots, spin qubits, spin-correlated radical pairs, spin polarization, electron paramagnetic resonance

## Abstract

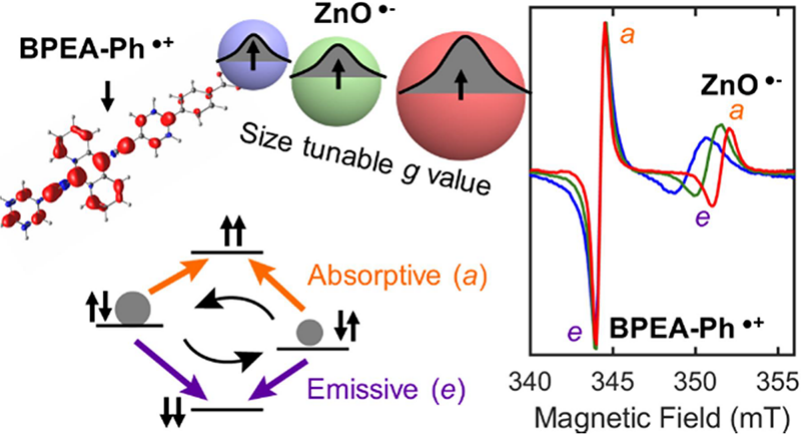

Organic molecules and quantum dots (QDs) have both shown promise as materials that can host quantum bits (qubits). This is in part because of their synthetic tunability. The current work employs a combination of both materials to demonstrate a series of tunable quantum dot–organic molecule conjugates that can both host photogenerated spin-based qubit pairs (SQPs) and sensitize molecular triplet states. The photogenerated qubit pairs, composed of a spin-correlated radical pair (SCRP), are particularly intriguing since they can be initialized in well-defined, nonthermally populated, quantum states. Additionally, the radical pair enables charge recombination to a polarized molecular triplet state, also in a well-defined quantum state. The materials underlying this system are an organic molecular chromophore and electron donor, 9,10-bis(phenylethynyl)anthracene, and a quantum dot acceptor composed of ZnO. We prepare a series of quantum dot–molecule conjugates that possess variable quantum dot size and two different linker lengths connecting the two moieties. Optical spectroscopy revealed that the QD–molecule conjugates undergo photoexcited charge separation to generate long-lived charge-separated radical pairs. The resulting spin states are probed using light-induced time-resolved electron paramagnetic resonance (TR-EPR) spectroscopy, revealing the presence of singlet-generated SCRPs and molecular triplet states. Notably, the EPR spectra of the radical pairs are dependent on the geometry of this highly tunable system. The *g* value of the ZnO QD anion is size tunable, and the line widths are influenced by radical pair separation. Overall, this work demonstrates the power of synthetic tunability in adjusting the spin specific addressability, satisfying a key requirement of functional qubit systems.

## Main Text

Identifying and characterizing new material systems that can host qubits is necessary to advance quantum information science (QIS) technologies.^1,2^ Both molecules^3−7^ and colloidal quantum dots (QDs)^8−10^ have drawn recent interest as bottom-up QIS building blocks since they offer considerable synthetic tunability. Many properties of colloidal QDs (e.g., optical and magnetic) can be synthetically tuned via composition,^11^ size,^12^ and morphology,^13^ and they also offer functionalizable surfaces onto which molecular species can be attached.^14^ Furthermore, colloidal QDs can be synthesized at scale,^15^ are amenable to solution-based processing,^16^ and can be self-assembled into ordered arrays.^17^ Molecular qubits also offer a striking degree of variety and tunability that has been harnessed to demonstrate gate operations,^18^ long decoherence times using clock states^19−22^ and isotopic control,^23,24^ and preparation of ordered arrays.^22,25^ QD – molecule conjugates are therefore an intriguing, yet underexplored, platform for hosting spin qubits.^26−29^ The current work takes advantage of the synthetic control of both QDs and molecules to demonstrate tunability within a spin qubit pair (SQP) on a QD – molecule conjugate.

Photogenerated SQPs offer unique advantages as components in QIS applications. Notably, they can be prepared in well-defined, non-Boltzmann populated, spin states at moderate temperatures. These states are generated when photoexcitation of a singlet state is rapidly followed by charge separation to yield a two-spin, four-state quantum system with selective population of two m_s_ = 0 states with singlet character. These states can be manipulated using microwave pulse sequences in electron paramagnetic resonance (EPR) spectrometers. Photogenerated SQPs, typically referred to as spin-correlated radical pairs (SCRP) in natural and artificial photosynthetic assemblies, have therefore drawn considerable attention for quantum computation^30^ and quantum sensing^31^ applications. Recent work on SQPs, primarily from the Wasielewski group, has demonstrated that SQPs can be used for spin state teleportation,^32^ CNOT gate operation,^33,34^ spin selective addressability,^35^ and chirality induced spin selectivity.^36,37^ Photogenerated SQPs can therefore satisfy the DiVincenzo criteria^38^ for functioning qubits: they are scalable, can be initialized in well-defined states, possess long decoherence times, are selectively addressable, and quantum gate operations can be performed.

Although the majority of work on photogenerated SQPs has relied on molecular hosts, it has recently been shown that QD – molecule conjugates can also serve as hosts of SQPs.^26−29,39^ Work from the Wasielewski and Sessoli groups found that photoexcitation of CdSe QDs could spin polarize appended molecules, possibly through a radical pair mechanism, but the QD radical was not detected due to spin decoherence on the heavy-atom containing QDs.^26,28^ In work from the Wu group, quantum beats between the singlet and triplet states in a SQP on a QD – molecule conjugate were observed by using ultrafast spectroscopy to optically probe recombination products.^29^ This work complements our approach, which employs EPR to probe the spin states of SQPs in QD – molecule conjugates.

In our prior work,^27^ we took advantage of the relatively long coherence times of electron spins in ZnO QDs (T_2_ ∼ 50 ns at room temperature),^40,41^ to provide the first direct EPR measurement of a SQP in which one spin is delocalized in a QD. Electron spins had previously been studied in ZnO QDs by using photodoping to prepare trapped delocalized electrons in the ZnO conduction band.^42,43^ The *g* value of these spins was found to be size tunable (due to quantum confinement), ranging from 1.96 to 1.97,^44−46^ notably distinct from the typical 1.99–2.01 *g* value range of organic molecules. To transiently prepare such spin states as part of a SQP, we photoexcited a well-known organic dye molecule, D131, attached to the surface of ZnO QDs to generate D131^•+^ – ZnO^•-^ SQPs. We observed light-induced highly polarized EPR signatures at magnetic fields associated with *g* = 2.003 (D131^•+^) and *g* = 1.962 (ZnO^•-^), consistent with a SQP.^27^

Molecular triplet excitations were not observed in our prior study, but prior research in organic-only systems have demonstrated that SQPs can be effective triplet sensitizers.^47−53^ Understanding and controlling spin dynamics in and sensitization of molecular triplet states has drawn recent interest within the context of singlet fission^54−56^ and triplet–triplet annihilation.^57,58^ Notably, QDs have been demonstrated as effective photosensitizers for molecular triplet states,^58,59^ relying on photoexcitation of the QD, followed by triplet–triplet energy transfer. Intersystem crossing (ISC) following direct photoexcitation of the chromophore of interest can also be achieved through radical pair intermediates.^47−53^ However, employing radical pairs in a QD – molecule conjugate to sensitize molecular triplet states has only been demonstrated with EPR in one prior report,^60^ and the radical pair in this system was not probed.

The current work expands on our prior study to demonstrate synthetic tunability of the SQP based on the QD size and the linker length between the organic chromophore and ZnO QD. This allows us to tune both the bandgap energy and thus the *g* value of the spin polarized ZnO^•-^ resonance (due to quantum confinement) as well as the dipolar and exchange coupling. We also show that the SQP can sensitize a molecular triplet state in well-defined spin configuration via radical pair ISC. The synthetic tunability of this system offers great potential in generating spectrally addressable spin-polarized systems that can serve as qubits.

## Results and Discussion

**Figure 1:**
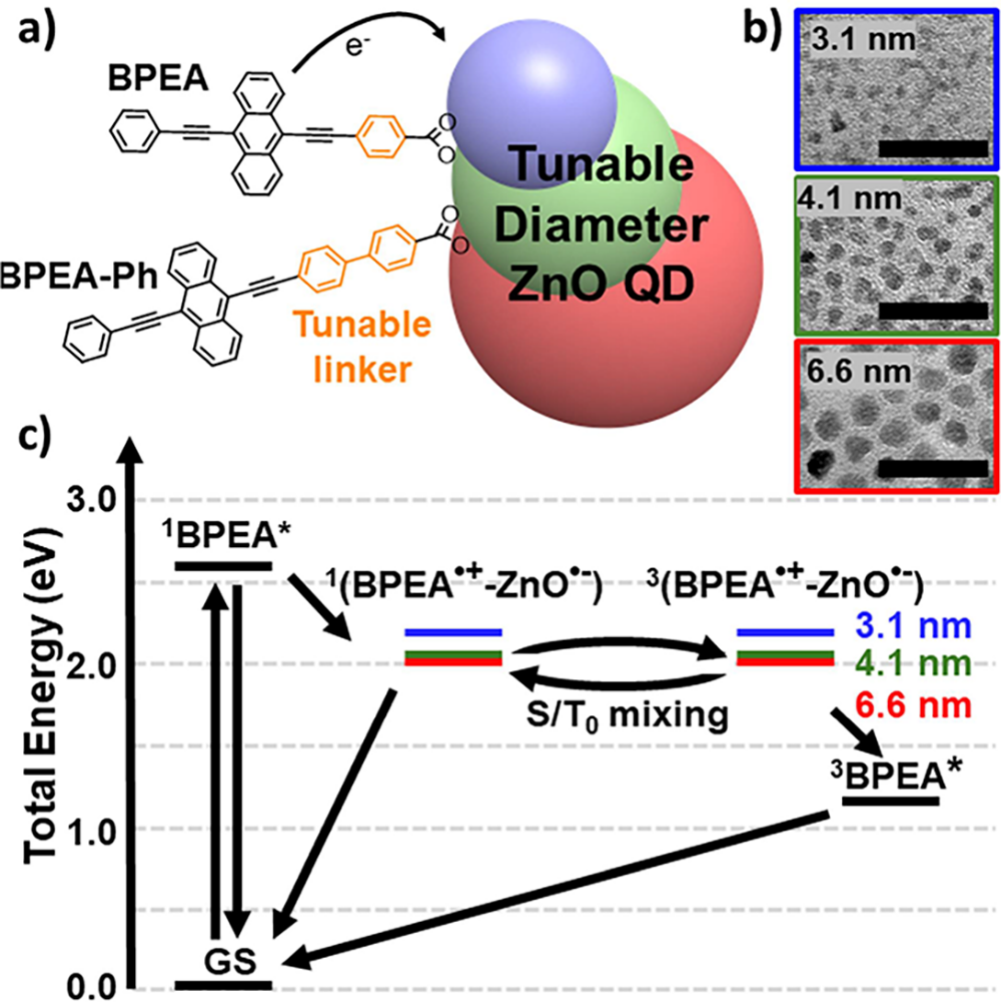
BPEA – ZnO QD Conjugates. a) Cartoon of dye – ZnO QD conjugates. b) Transmission electron microscopy (TEM) micrographs of ZnO QDs with 30 nm scale bar. c) Predicted Jablonski diagram for the BPEA – ZnO QD system summarizing charge separation and recombination pathways (BPEA-Ph – ZnO QD energetics are very similar, see Table S3). Ion pair energies are depicted for 3.1 nm (blue), 4.1 nm (green), and 6.6 nm (red) ZnO QDs.

We prepared a series of QD – molecule conjugates composed of a 9,10-bis(phenylethynyl)anthracene chromophore and electron donor, a variable length organic linker, and ZnO QD electron acceptors of three different sizes (Figure 1a). The anthracene-based donors are expected to host radical cations that can serve as molecular qubits (with long spin coherence) as has been shown in prior anthracene-based systems.^7^ These organic molecules, BPEA and BPEA-Ph, acting both as chromophore and linker, were synthesized using established organic synthetic approaches.^61−63^ ZnO QDs of three sizes were synthesized,^64^ and their diameters determined with electron microscopy to be 3.1 ± 0.5 nm, 4.1 ± 0.6 nm, and 6.6 ± 1.2 nm (Figure 1b). QD extinction coefficients were determined using optical absorption spectra and standard sizing curves.^65,66^ We found that the ZnO QDs were susceptible to Ostwald ripening^67^ unless stored dry and in a freezer. QD – molecule conjugates were then prepared by mixing stoichiometric ratios of QDs and BPEA derivatives in tetrahydrofuran.

Photoexcitation of the BPEA moiety within these conjugates can initiate electron transfer to produce the BPEA^•+^ – ZnO^•-^ radical pair (Figure 1c). Ion pair energies were approximated with the Weller expression,^68^ using the BPEA oxidation potential (*E*_ox_ = −1.21 V vs SCE),^69^ the reduction potential of bulk ZnO (E_red_ = −0.55 V vs SCE),^70^ and radical pair separations based on the estimated centers of the delocalized ions, r_DA_. Size-dependent reduction potentials of the ZnO QDs were calculated based on bandgap changes, and were consistent with electrochemical studies on ZnO QDs.^71^ Detailed calculations and ion pair values can be found in the Supporting Information (SI) (Table S3). Figure 1c further illustrates the mixing of the singlet and triplet radical pair that is a hallmark of SCRPs, and facilitates recombination to the BPEA triplet state, which lies 1.2–1.3 eV above the ground state, but 0.8–1.0 eV below the charge-separated radical pair state.^72^

**Figure 2:**
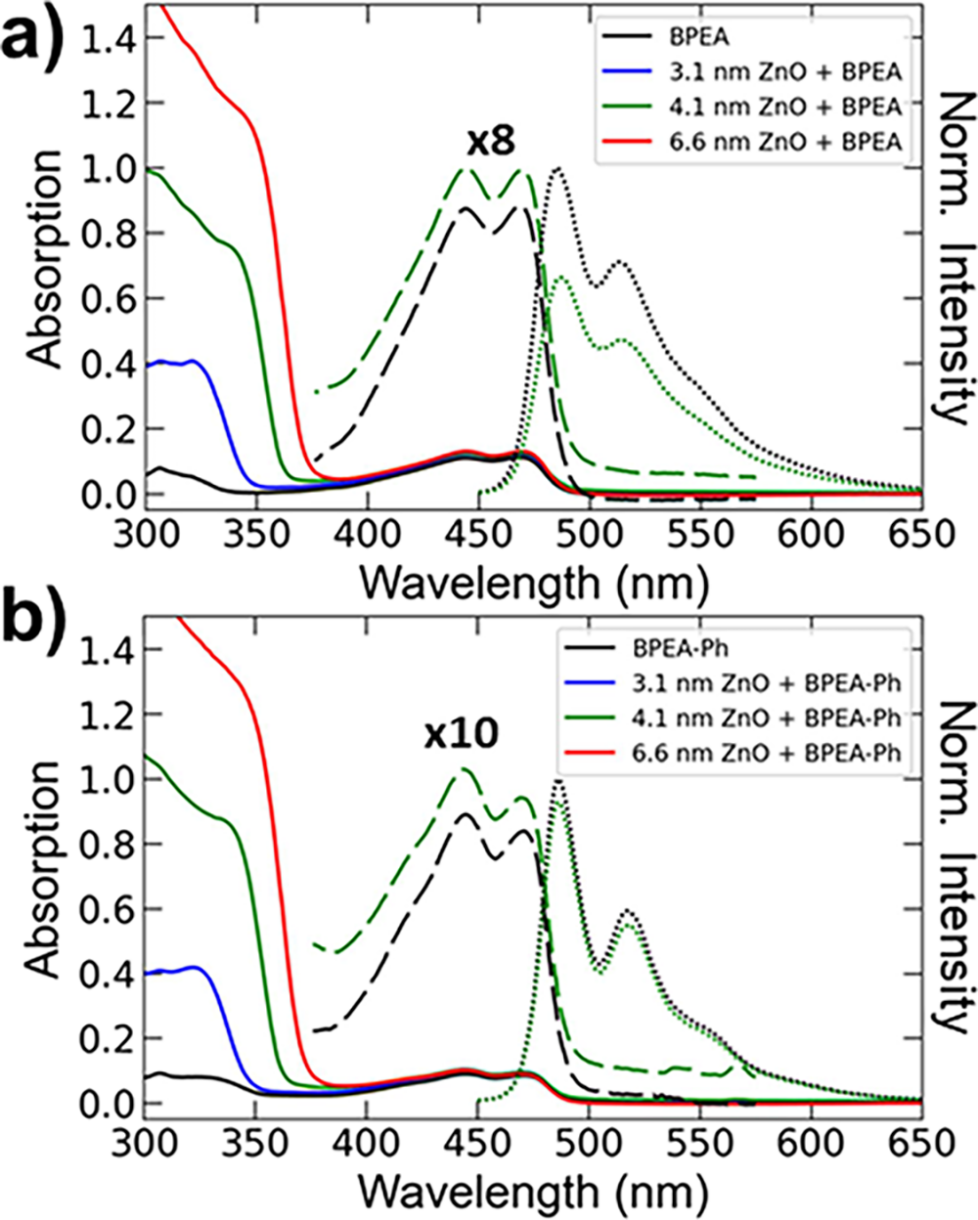
Steady state optical characterization of (a) BPEA and (b) BPEA-Ph with ZnO QDs. Absorption spectra of BPEA (BPEA-Ph) (black) and BPEA – ZnO (BPEA-Ph – ZnO) conjugates with varying size ZnO QDs (3.1 nm in blue, 4.1 nm in green, 6.6 nm in red) and ∼ 2 BPEA (BPEA-Ph) per QD are shown in a (b). Fluorescence spectra of BPEA (BPEA-Ph) (black dotted) and BPEA – ZnO (BPEA-Ph – ZnO) conjugates with ∼ 2 BPEA (BPEA-Ph) per 4.1 nm ZnO QDs (green dotted) are also overlaid in a (b). All measurements were performed at room temperature in tetrahydrofuran.

With an understanding of the general energetics of this system, we turned to optical experiments to characterize BPEA – ZnO QD interactions and photoexcited charge dynamics. Steady-state absorption spectra are shown in Figure 2 for the BPEA – ZnO QD (and BPEA-Ph – ZnO QD) conjugates with three different QD sizes, showcasing the increasing bandgap with decreasing QD size. Emission spectra of BPEA (and BPEA-Ph) revealed modest fluorescence quenching of both BPEA (∼33% reduction) and BPEA-Ph (∼5–10% reduction) upon addition of ZnO QDs of all three sizes (see Figures S3–S5 for details). This observation suggests that either the binding of BPEA to ZnO QDs is inefficient or that light-induced electron transfer is comparable to radiative recombination, and that this effect is more pronounced for BPEA-Ph than for BPEA.

**Figure 3:**
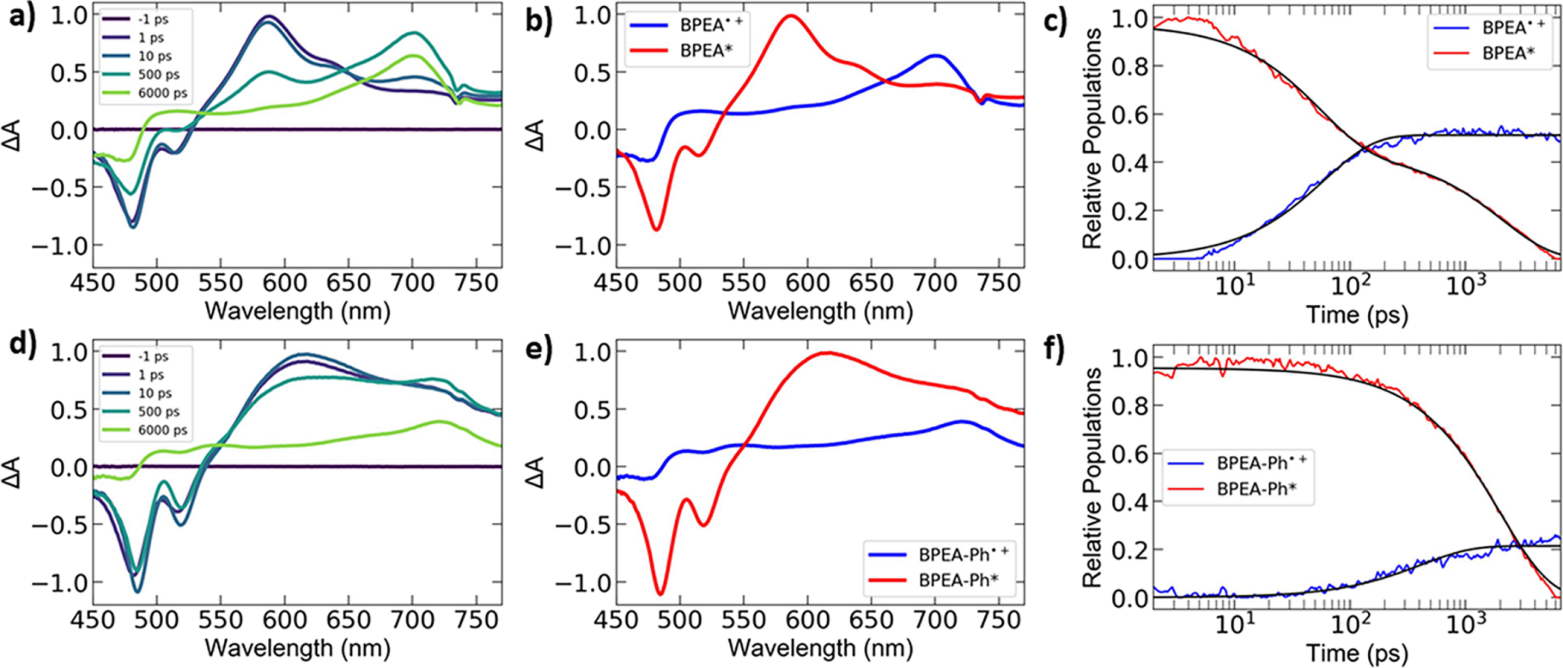
Femtosecond transient absorption spectroscopy for BPEA – ZnO QD (5.8 nm) and BPEA-Ph – ZnO QD (5.8 nm) conjugates in toluene at room temperature, excited at 420 nm. a, d) Transient spectra at different times for BPEA – ZnO and BPEA-Ph – ZnO samples, respectively. b, e) Basis spectra of the long-lived BPEA^•+^ (or BPEA-Ph^•+^) state (blue) and BPEA* (or BPEA-Ph*) (red) used to deconvolve the transient spectra. c, f) Time traces of BPEA* (or BPEA-Ph*) populations (red), BPEA^•+^ (or BPEA-Ph^•+^) populations (blue), and kinetic fits (black) used to determine charge transfer kinetics.

Femtosecond transient absorption experiments were performed to understand BPEA and BPEA-Ph binding to ZnO QDs and the photoexcited dynamics of the conjugates. The excitation wavelength was 420 nm, which results exclusively in excitation of BPEA and BPEA-Ph and not the ZnO QD (Figure 2). Transient spectra are shown in Figure 3a and 3d, and exhibit stimulated emission (negative peaks at ∼ 480 and 520 nm), excited state absorption (BPEA* or BPEA-Ph*, ∼ 590 nm), and BPEA radical cation absorption (BPEA^•+^ or BPEA-Ph^•+^, ∼ 700–720 nm).^69^ We do not expect to observe absorption associated with intraband transitions in ZnO^•-^, since these occur in the IR.^40,73^ To deconvolve the overlapping spectral signatures, basis spectra were chosen at 5 ps (BPEA* or BPEA-Ph*) and 6000 ps (BPEA^•+^ or BPEA-Ph^•+^). It should be noted that the excited state basis spectra also include stimulated emission (with the same time constant), and the cation basis spectra include features at 450 nm that we assign to a BPEA triplet.^74^ Spectral fits using these basis spectra were performed for each time point to predict relative populations of BPEA* (or BPEA-Ph*) and BPEA^•+^ (or BPEA-Ph^•+^) in time, which were then fit to exponential functions (Figure 3c,f). Further details are provided in the Methods section, Figure S7, and Table S4.

Analysis of the relative populations of BPEA* (BPEA-Ph*) and BPEA^•+^ (BPEA-Ph^•+^) in time can help to clarify questions related to binding and charge transfer. We found that BPEA* decayed biexponentially with time constants (relative amplitudes) of 54 (0.55) and 2080 (0.45) ps, while the BPEA^•+^ cation grew in with a time constant of 59 ps. Therefore, we conclude that a subpopulation of BPEA molecules undergo ∼ 54–59 ps charge transfer, while another population decays radiatively. This is corroborated by the fact that BPEA and BPEA-Ph not bound to ZnO QDs have radiative lifetimes of 2–3 ns (Figure S6), and from the steady-state fluorescence (Figure 2a) in which BPEA fluorescence was only partially suppressed upon ZnO addition. We note that there is a discrepancy in steady state fluorescence experiments in which ∼ 33% of BPEA undergoes charge transfer to the QDs, while the transient absorption experiments indicate that 55% of BPEA undergoes charge separation. This is likely a result of different solvents for the two experiments: tetrahydrofuran for steady state quenching since it solubilizes BPEA, and toluene for transient absorption to better match EPR experiments. The BPEA-Ph* decay (Figure 3f) was entirely dominated by radiative recombination (time constant of 2010 ps) and the BPEA-Ph steady-state fluorescence was only marginally (∼10%) quenched by ZnO addition (Figure 2b). These observations indicate that a small fraction (5–20%) of BPEA-Ph was able to facilitate charge separation. Nevertheless, this small population did produce detectable BPEA-Ph^•+^ cation, which appeared with a 410 ps time constant. A relative population of 20% was assigned to the BPEA-Ph^•+^ with the assumption that it has a similar extinction coefficient to BPEA^•+^. The longer time constant of charge separation for BPEA-Ph relative to BPEA is consistent with the longer linker length and corresponds to a tunneling coefficient of β = 0.5 Å^–1^. This is well within the normal range for electron tunneling through oligophenyl groups,^75^ thus validating a vertically attached geometry for the chromophores on the ZnO QD surface.

To summarize the results from the optical experiments, we found that photoexcited charge separation occurs from BPEA (∼55 ps) and BPEA-Ph (∼410 ps) to ZnO QDs to generate BPEA^•+^ – ZnO^•-^ (and BPEA-Ph^•+^ – ZnO^•-^) radical pairs that persist for >8 ns (maximum pump–probe delay). However, only 30–55% of BPEA and 5–20% of BPEA-Ph undergoes charge separation upon photoexcitation. Note, there is some variability in these percentages due to different solvents. We hypothesize that inefficient binding of BPEA and BPEA-Ph molecules to the ZnO QD surface explains the inefficient charge separation. Prior work has shown that Zn^2+^ ions that are present in the ZnO QD solutions can competitively bind to carboxylate containing molecules like BPEA and BPEA-Ph, and therefore limit binding to the QDs themselves.^64,76−78^ This model appears to offer an accurate explanation for our observations since the Stern–Volmer quenching curves (Figure S5), qualitatively match what has been seen in the literature.^76^ The dynamic nature of the binding limits our ability to isolate pure QD – molecule conjugates without free BPEA or BPEA-Ph. However, the presence of spectroscopically relevant quantities of photogenerated BPEA^•+^– ZnO^•-^ (and BPEA-Ph^•+^ – ZnO^•-^) radical pairs was sufficient to move forward with transient EPR measurements on these tunable radical pairs.

**Figure 4:**
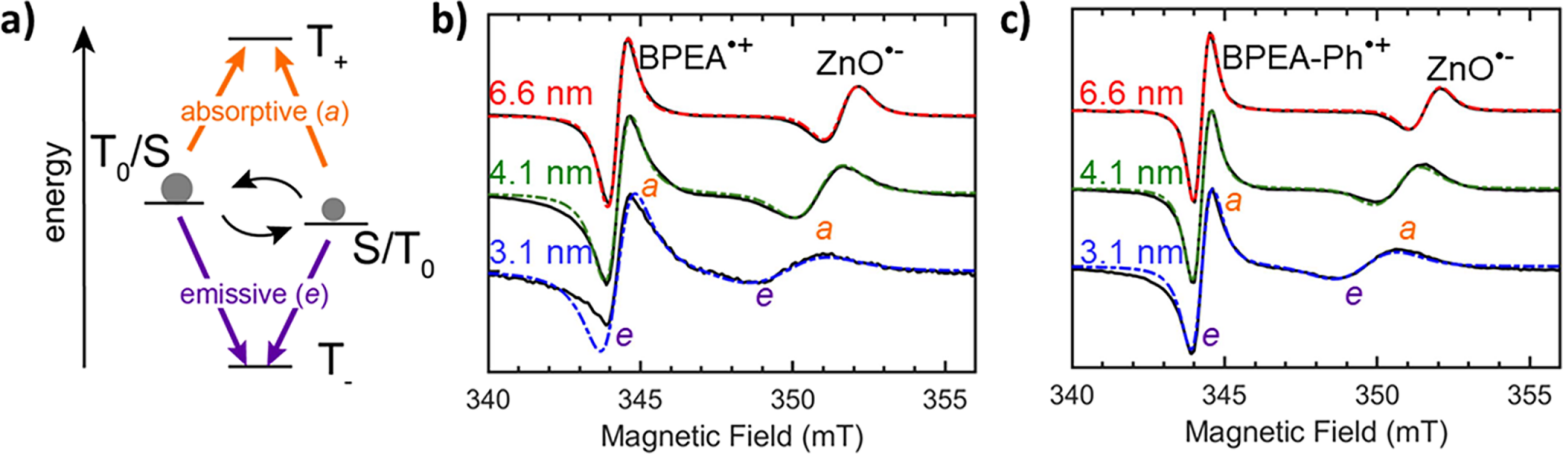
TR-EPR of photogenerated spin-correlated radical pairs (SCRP). a) Energetics of SCRP highlighting mixing between m_s_ = 0 levels that give rise to two absorptive (*a*) and two emissive (*e*) transitions. TR-EPR data for BPEA – ZnO (BPEA-Ph – ZnO) QD conjugates with varying QD size are shown in b (c). Experimental spectra (black lines) were collected in frozen (20 K) toluene and spectra were averaged over 600–1500 ns after excitation with a 450 nm pulsed laser. Simulations (colored dashed lines) are shown for the different ZnO diameters, blue (3.1 nm), green (4.1 nm), and red (6.6 nm).

The photogenerated spin states were investigated with transient EPR (TR-EPR) spectroscopy which uses weak continuous microwave irradiation without field modulation.^79,80^ The spectra shown were obtained following laser excitation and then analyzed within the context of the SCRP model.^30,79,81−83^ In the SCRP model, the significant spatial separation of unpaired electron spins reduces the exchange coupling (*J*) and dipolar coupling (*d*) such that mixing can occur between the singlet and triplet manifolds. In the presence of a large external magnetic field (here: ≈0.3 T), the energy levels of the T_+_ and T_–_ (m_s_ = ± 1) states of the radical pair (Figure 4a, Figure S10) are far above and below the energy levels of the T_0_ and S states (m_s_ = 0), respectively. Therefore, only the T_0_ and S states can mix to produce two states with both singlet and triplet character. Mixing is facilitated by a difference in *g* values of the respective radicals and hyperfine interactions. Since charge separation starts from the photoexcited singlet state of the electron donor BPEA (or BPEA-Ph) and is faster than any spin dynamics in the radical pair(s), the SCRP initially maintains singlet character. The result is a four-state quantum system in which the two middle states are populated, while the T_+_ and T_–_ remain unpopulated. This non-Boltzmann populated, spin-polarized, system can then be probed with TR-EPR. Since no field modulation with lock-in detection is used, the spectra appear not as first derivative type but as directly detected absorption-emission spectra. An SCRP is expected to yield four transitions, two emissive (*e*) and two absorptive (*a*) signals for each orientation of the molecule with respect to the magnetic field.

TR-EPR spectra averaged 600–1500 ns after pulsed laser (450 nm) excitation are shown for BPEA^•+^ – ZnO^•-^ and BPEA-Ph^•+^ – ZnO^•-^ radical pairs with three ZnO QD sizes in Figure 4b and 4c, respectively. The spectra of all six samples exhibit an *e, a, e, a* polarization pattern, indicative of a singlet-generated SCRP where the T_0_ state is lower in energy than the singlet state. As expected, two transitions are centered at the *g* value associated with BPEA^•+^ (≈2.003), close to free electron *g* value, and the other two are centered at the *g* value of a ZnO^•-^ QD (1.96 −1.97). Radical pair simulations were performed including the following parameters:^84,85^*J*, *d*, the *g* values of ZnO^•-^ and BPEA^•+^ (or BPEA-Ph^•+^), the line widths (expressed as both a *g* strain and Lorentzian broadening), and the relative contributions from T_0_ and S. Some parameters were constrained in accordance with our physical understanding of the system. The dipolar coupling was determined by the radical pair spacing (, see Equation S9). The exchange coupling was fixed to decay exponentially with radical pair spacing (*J* ∝ *e*^–β*r*_*DA*_^),^86^ with the value of J for the sample with the smallest value or r_DA_ as an adjustable parameter. The contribution from T_0_ was found to be below 5%, which is expected since these radical pairs are produced from a singlet excited state. However, some spin evolution may occur during the charge transfer process. Other parameters were adjusted to improve correspondence between simulations and experiment (see Table S5 for all parameters).

**Figure 5:**
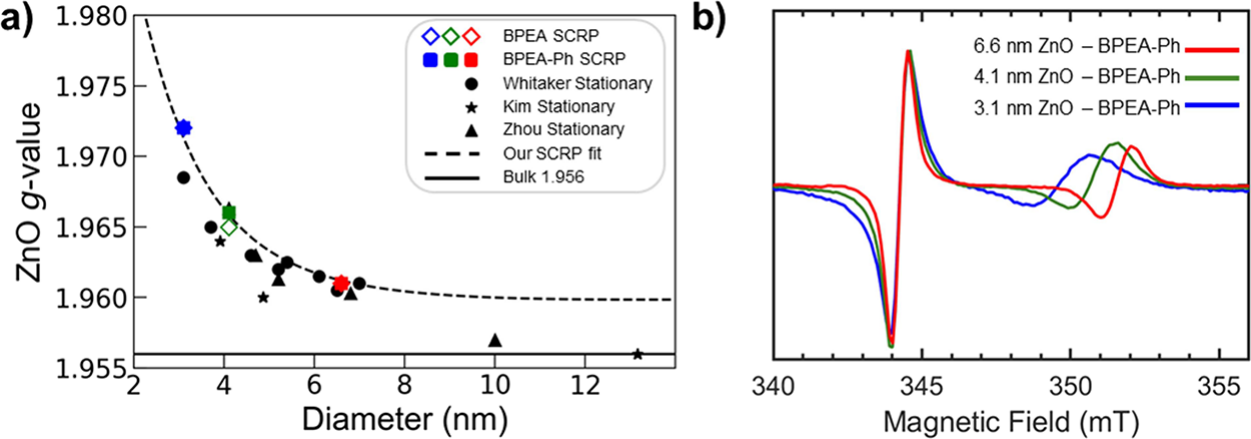
a) ZnO *g* value as a function of QD diameter based on our radical pair data (blue = 3.1 nm, green = 4.1 nm, and red = 6.6 nm), fit to eq 1. For reference, data on stable ZnO QD anions from Whitaker et al.,^41^ Kim et al.,^43^ and Zhou et al.^71^ are also shown. b) Overlay of all SCRP data for BPEA-Ph to highlight both the size-dependence of the ZnO^•-^*g*-value as well as the changes in line width for the BPEA-Ph^•+^.

The simulations allowed us to quantify an important trend in the data: the size-dependence of the ZnO^•-^*g* value. This trend is qualitatively apparent in Figure 4, since the ZnO^•-^ resonance shifts to higher magnetic field (smaller *g* value) with increasing QD size, slowly approaching the value for bulk ZnO, 1.956.^44,87^ This relationship has previously been observed for photochemically prepared trapped spins in colloidal ZnO QDs.^44,46,88^ Whitaker et al.^44^ and Zhou et al.^88^ used temperature-dependent measurements to show that these unpaired spins were delocalized in conduction band like states (i.e., the 1S_e_ state of a spherical ZnO QD). Furthermore, they found that the size-dependence of the *g* value could be modeled by eq 1:^44,88^
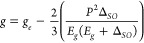
1where *g*_*e*_ = 2.0023 is the *g* value of a free electron, *P*^2^ is the interband matrix element (set to 20 eV, in accordance with prior work),^44^*E*_*g*_ is the bandgap of the ZnO QDs, and Δ_SO_ is the spin–orbit coupling constant. We fit our extracted *g* values from the radical pair simulations to eq 1 (Figure 5a), using an experimental sizing curve to determine *E*_*g*_,^66^ and allowing Δ_SO_ to be the fitting parameter. We extracted a value of Δ_SO_ = 36 meV, similar to the value of 40 meV found by Whitaker et al.^44^ EPR data from prior reports on stable (stationary) spins in ZnO QDs are also shown in Figure 5a, showing the similarity between our photogenerated ZnO^•-^ and the photochemically prepared stable ZnO^•-^. This further confirms that the unpaired electron in the ZnO QD of photogenerated BPEA^•+^ – ZnO^•-^ (and BPEA-Ph^•+^ – ZnO^•-^) radical pair is in a delocalized QD conduction band like state that is subject to quantum confinement.

Another trend worth discussing is the effect of ZnO QD size and linker length on the observed line width. The line width of the ZnO^•-^ peak decreases as QD diameter increases (Figure 5b), which we attribute to the steeper slope of the *g* value vs diameter curve for smaller QD diameters. We can confirm this hypothesis by using the size distributions obtained from TEM to predict a *g* distribution based on eq 1. The predicted *g* value distributions (as fwhm) are 0.0095, 0.006, and 0.0024 (for small, medium, and large QDs). These present the same trend as the *g* strain values (fwhm) employed to fit the data: 0.012, 0.008, and 0.0055 (Figure S11, Table S6). Note that the extracted *g* strain values are systematically larger by about 0.003 (≈15 MHz at X-band) than is predicted by the size distribution. This small deviation is likely a result of intrinsic broadening in the ZnO^•-^, possibly from hyperfine interactions or lifetime broadening. The BPEA^•+^ and BPEA-Ph^•+^ line widths also decrease with increasing QD size and are narrower for BPEA-Ph than BPEA for the same QD size (Figure 4 and 5b). Since both organic cations exhibit minimal *g* anisotropy and similarly small, unresolved hyperfine couplings (Table S9 and S11), there must be another explanation for the trend in line width. Dipolar coupling () is one possible mechanism for line broadening. Although our simulations predict a minimal impact from *d*, it is possible that a distribution of dye configurations may create subpopulations with larger values of *d* that effectively increase the line width of the organic dye as the average radical pair separation decreases. Lifetime (homogeneous) broadening is another potential contributor to this trend in line widths whereby conjugates with shorter radical pair separation exhibit shorter lifetimes. Of note, our simulations capture the varying BPEA^•+^ and BPEA-Ph^•+^ line widths by changing the values for the Lorentzian broadening. The predicted values of T_2_ based on these fits range from 36 ns for the 3.1 nm – BPEA sample to 119 ns for the 6.6 nm - BPEA-Ph sample (Table S5), consistent with prior studies.^41^ However, the Lorentzian line widths may be an overestimate (and the associated T_2_’s an underestimate), due to inhomogeneous broadening (such as a distribution of dipolar couplings). We currently do not have enough information to confidently quantify the factors contributing to distance-dependent broadening trends.

**Figure 6:**
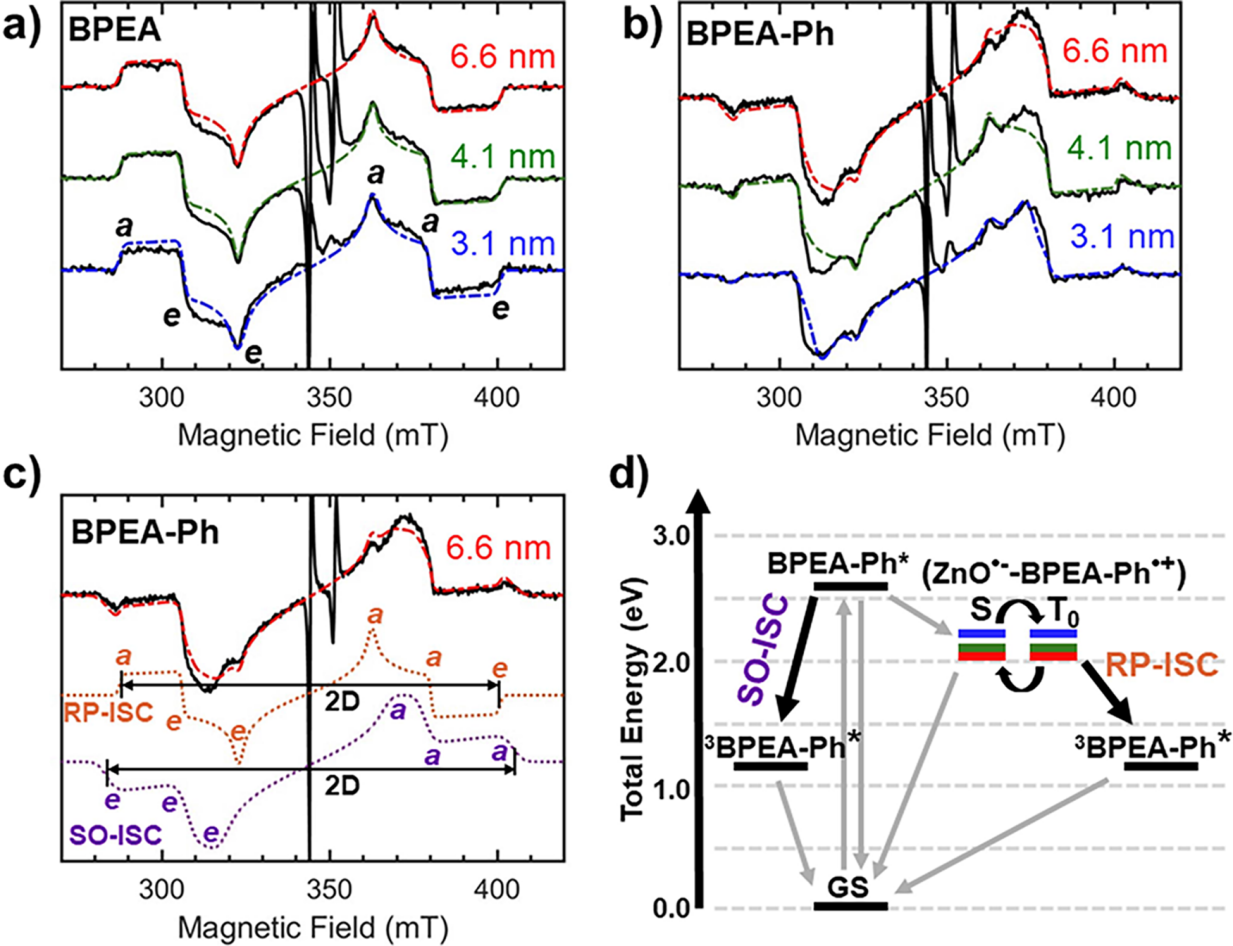
TR-EPR of photoexcited dye – ZnO conjugates with a wider magnetic field range, showing the presence of a BPEA triplet state. TR-EPR data for BPEA – ZnO (BPEA-Ph – ZnO) QD conjugates with varying QD size are shown in a (b). Experimental spectra (black lines) were collected in frozen (20 K) toluene and spectra were averaged over 400–1000 ns after excitation with a 450 nm pulsed laser. Simulations (colored dashed lines) are shown for the different ZnO diameters, blue (3.1 nm), green (4.1 nm), and red (6.6 nm). c) Example of the two species (RP-ISC, orange, and SO-ISC, purple) contributing to the full spectrum for the 6.6 nm BPEA-Ph sample d) Energy diagram highlighting the two potential pathways to generate the BPEA-Ph triplet (SO-ISC and RP-ISC).

We have so far shown that TR-EPR measurements on the BPEA^•+^ – ZnO^•-^ (and BPEA-Ph^•+^ – ZnO^•-^) radical pairs reveal geometry-dependent trends. Using this technique, we were also able to observe the photogenerated triplet states ^3^BPEA* and ^3^BPEA-Ph*. As shown in Figure 1, the triplet state is energetically accessible via energy transfer from either the excited singlet state (BPEA* and BPEA-Ph*) or recombination of the radical pair states ^3^(BPEA^•+^ – ZnO^•-^) and ^3^(BPEA-Ph^•+^ – ZnO^•-^). The former process is termed spin–orbit intersystem crossing (SO-ISC), while the latter process is mediated by a radical pair recombination and is thus termed radical pair intersystem crossing (RP-ISC). RP-ISC is also commonly referred to as the S-T_0_ or the electron back recombination (EBR) mechanism of triplet formation.^47−53^ Triplets generated by RP-ISC are preferentially populated in the T_0_ state due to the S-T_0_ mixing of the RP, and are expected to produce either an *a*, *e*, *e*, *a*, *a*, *e* or *e, a, a, e, e, a* polarization pattern, depending on the sign of the zero-field splitting (ZFS) parameter D.^47−50^ The BPEA – ZnO conjugates produce a triplet spectrum with exactly the *a*, *e*, *e*, *a*, *a*, *e* pattern, and it can be simulated assuming exclusive T_0_ population and positive ZFS parameter D (Figure 6a). The positive D parameter is also supported by DFT calculations (Table S10). The BPEA-Ph– ZnO conjugates, however, produce a triplet spectrum that can only be simulated as a combination of ∼ 50% RP-ISC and ∼ 50% SO-ISC (Figure 6b,c, Figure S14). Note that the ZFS parameters of the RP-ISC triplet are very similar both in BPEA (D ≈ 1589 MHz, E ≈ −157 MHz) and BPEA-Ph (D ≈ 1595 MHz, E ≈ −161 MHz), indicating that the localization of the triplet exciton is virtually identical in both molecules (Tables S10 and S12; Figures S17 and S20). However, the ZFS parameters of the SO-ISC triplet in BPEA-Ph are slightly different (D ≈ 1680 MHz, E ≈ −100 MHz). We can rationalize these observations as a consequence of the large percentage (>90%) of BPEA-Ph that does not undergo charge separation since it is not bound to ZnO QDs (see discussion on optical spectroscopy above) and thus experiences a slightly different molecular surrounding than BPEA-Ph bound to the ZnO QD. Overall, the presence of significant RP-ISC triplets in both samples suggests that these types of QD – molecule conjugates may be a useful material for efficiently sensitizing triplet states using light.

## Conclusions

We have employed synthetic tunability to explore the role of geometry on photogenerated SQPs/SCRPs within a series of QD – molecule conjugates. The spin states in this class of materials have only recently begun to be measured and probed by TR-EPR spectroscopy,^26,27^ and this work highlights the potential for using synthesis to rationally tune their spin properties. Standard synthetic techniques were used to prepare a series of ZnO QD – molecule conjugates with varying QD size and varying linker length. Upon photoexcitation of the molecular moiety (BPEA), we show that these conjugates generate spin-correlated radical pairs BPEA^•+^ – ZnO^•-^ and BPEA-Ph^•+^ – ZnO^•-^. Analysis of the light-induced time-resolved EPR spectra for these radical pairs allowed us to identify geometry-dependent trends in the data. Notably, the *g* value of the ZnO^•-^ resonance within the radical pair is QD size-dependent, enabling spectral tunability. Additionally, the line widths increase with decreasing radical pair separation, which may be a result of a combination of increasing lifetime broadening and dipolar interactions for smaller radical pair separations. These geometry-specific trends may help aid in the design of QD – molecule systems that can host qubit pairs with longer coherence times and precise spectral control. Finally, the radical pair state was also found to efficiently sensitize molecular triplet states via radical pair ISC, the second such demonstration using QD – molecule conjugates.

## Methods

### Synthesis

#### Chemicals

9,10-dibromoanthracene (>98%), iodine (I_2_) (>98%), triphenylphosphine palladium (0) (>97%), copper(I) iodide (>98%), sodium chloride (>99.5%), magnesium sulfate (>98%), trimethylsilylacetylene (>98%), hydrochloric acid (1M) triethylamine (>99%), silica gel, Bis(triphenylphosphine)palladium(II) dichloride (>98%), 4-Bromo-4’-iodo-1,1’-biphenyl (>98%), potassium vinyltrifluoroborate (>97%) and cesium carbonate (>98%) were purchased from TCI America. Zinc acetate (99.99% trace metals basis), *n*-butyl lithium (1.6 M in THF), sodium thiosulfate (99.99% trace metals basis), phenylacetylene (98%), diisopropylamine (DIPA, ≥ 99%), 4-Bromostyrene (97%), cesium fluoride (≥98%), triphenylphosphine (99%), polyethylene glycol, osmium tetroxide (2.5 wt % in *tert*-butanol), *tert*-butanol (≥99%), dimethylformamide (DMF, anhydrous, 99.8%), dimethyl sulfoxide (DMSO, anhydrous, ≥ 99.9%), ethyl alcohol (anhydrous, ≥ 99.5%), ethyl acetate (reagent, ≥ 99.5%), and oxone were purchased from Sigma-Aldrich. Tetramethylammonium hydroxide pentahydrate (TMAH, 98%) and oleic acid (OLA, tech., 90%) were purchased from Alfa Aesar. Solvents used were hexanes, acetone, toluene, tetrahydrofuran (THF), ethanol, methanol, anisole, *tert*-butanol, deionized water, dimethylformamide (DMF), and dichloromethane (DCM).

#### Synthesis of BPEA and BPEA-Ph

**Scheme 1:**
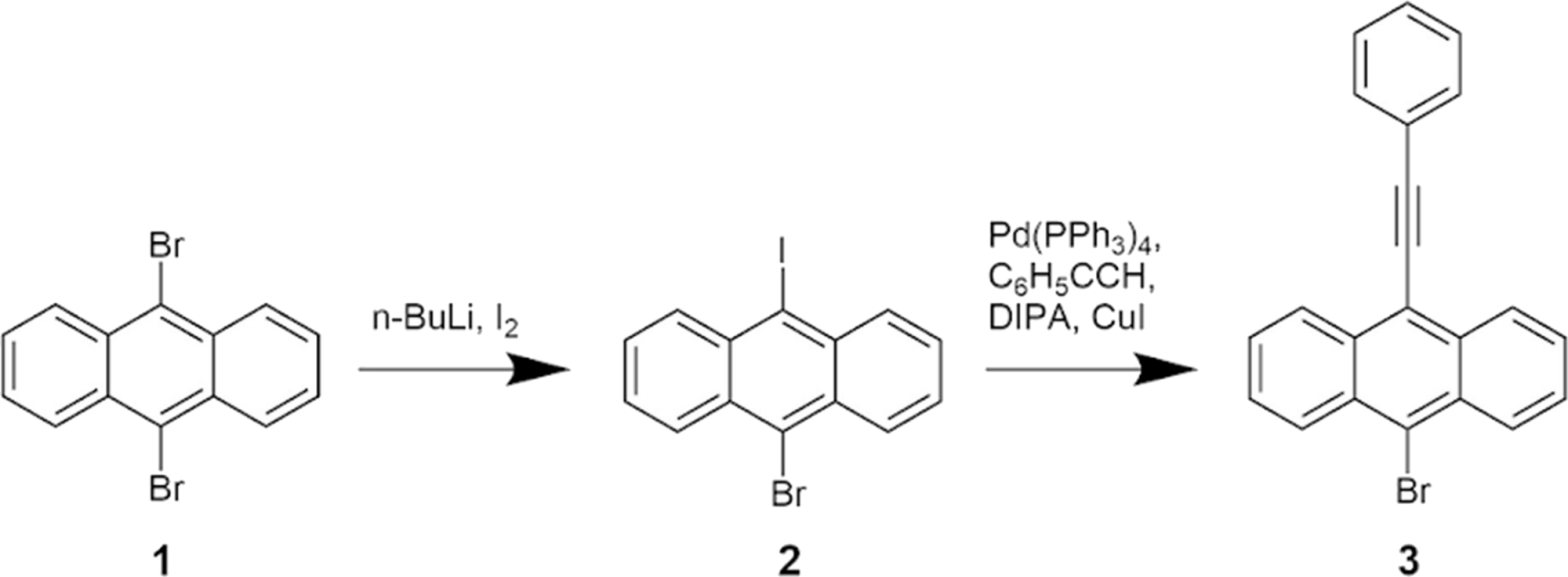
Schematic of the Synthesis of Reagent **3**

Synthesis of reagent **2** (Scheme 1): 9,10-dibromoanthracene (reagent **1**, 5 g), 100 mL THF and a stir bar were added to a flask, placed under N_2_, and cooled to −78 °C. Then 10.05 mL *n*-butyl lithium (1.6 M in THF) was added dropwise over 3 h. After the addition, I_2_ (4.89 g) in 25 mL THF was added dropwise and the solution was stirred overnight. Na_2_S_2_O_3_ was added until no color change occurred anymore. Then deionized water was poured into the suspension and the crude product was vacuum filtered and washed with cold ethanol. The filtered yellow residue was recrystallized in toluene to give product **2** with a yield of 4.38 g, yield: 89%. ^1^H NMR (CDCl_3_): δ = 8.64–8.52 (m, 4H), 7.68 – 7.56 (m, 4H) ppm.

Synthesis of reagent **3** (Scheme 1): Reagent **2** (658 mg), phenylacetylene (175 mg), and tetrakis(triphenylphosphine)palladium(0) (39.3 mg) were added to a solution of 7.67 mL toluene and 3.29 mL DIPA. The suspension was bubbled with N_2_ for 20 min and copper(I) iodide (6.6 mg) was added. The suspension was stirred and refluxed at 55 °C for 24 h. The final crude was extracted and washed with deionized water three times, then brine and MgSO_4_, filtered and recrystallized in toluene to obtain pure product **3**.^62^ Yield: 51%. ^1^H NMR (CDCl_3_): δ = 8.69 (m, 2H), 8.57 (m, 2H), 7.77 (m, 2H), 7.63 (m, 4H), 7.45(m, 3H) ppm.

**Scheme 2:**
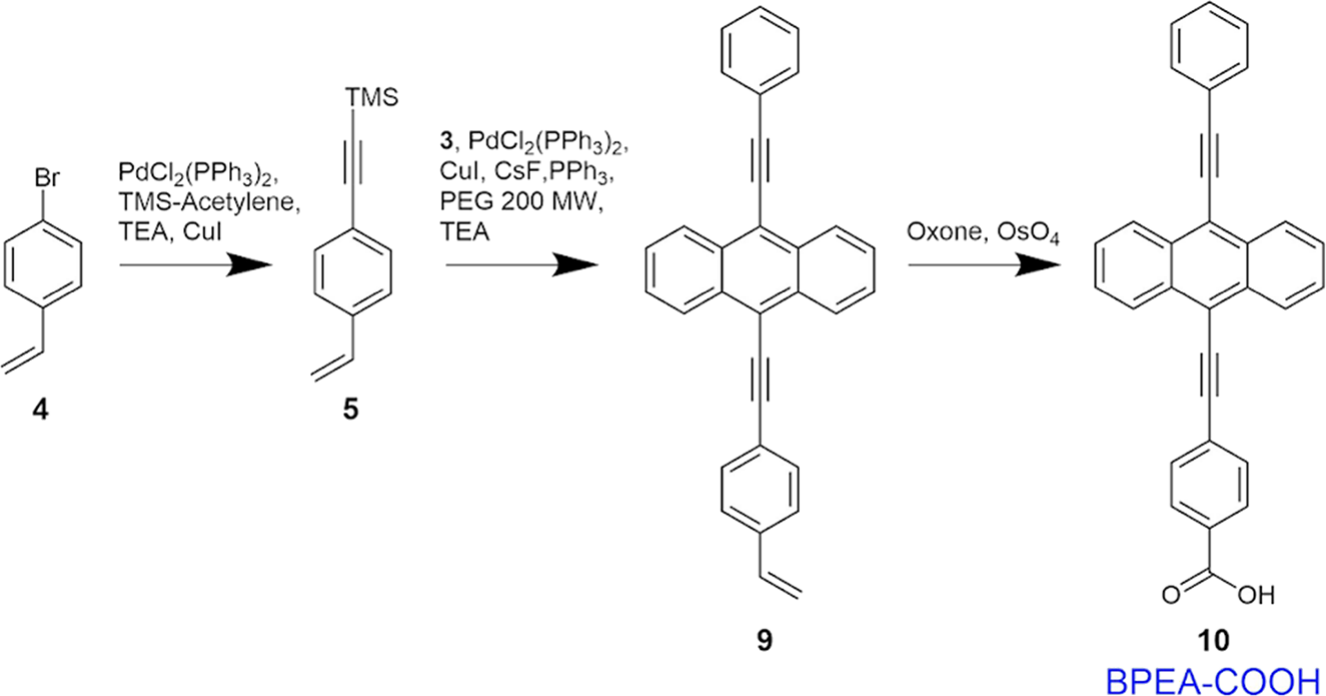
Schematic of the Synthesis of Reagent **10** (BPEA-COOH, Termed BPEA in the Results and Discussion)

Synthesis of reagent **5** (Scheme 2): In a dark environment, 4-bromostyrene (reagent **4**, 1.86 g), trimethylsilylacetylene (2.28 g), bis(triphenylphosphine)palladium(II) dichloride (0.14 g), and triethylamine (40 mL) were combined and stirred under N_2_. Copper(I) iodide (38 mg) was then added and the solution was bubbled for 15 min. After 15 min, the suspension was stirred and refluxed at 50 °C for 16 h. The final crude was evaporated and chromatographed on silica gel with hexane to obtain pure product **5**, yield: 54%. ^1^H NMR (CDCl_3_): δ = 7.44 (d, 2H), 7.35 (d, 2H), 6.69 (dd, 1H), 5.77 (d, 1H), 5.30 (d, 1H), 0.28 (s, 9H) ppm.

Synthesis of reagent **9** (Scheme 2): Reagent **3** (320.9 mg), copper(I) iodide (10.3 mg), cesium fluoride (273 mg), bis(triphenylphosphine)palladium(II) dichloride (18.9 mg), and triphenylphosphine (14.1 mg) were added to a 25 mL flask under N_2_. Then a mixture of reagent **5** (270 mg), polyethylene glycol (0.32 mL), triethylamine (2.89 mL) and deionized water (0.16 mL) were injected into the 25 mL flask. The suspension was bubbled with N_2_ for 15 min and stirred at 80 °C for 16 h. The final crude was extracted and washed with deionized water three times, then brine and MgSO_4_, filtered and dried by rotary evaporator to obtain 314 mg product **9**.^61^ Yield: 86%. ^1^H NMR (CDCl_3_): δ = 8.71 (m, 4H), 7.79 (d, 2H), 7.75 (d, 2H), 7.65 (m, 4H), 7.46 (m, 5H), 6.77 (dd, 1H), 5.87 (d, 1H), 5.37 (d, 1H) ppm.

Synthesis of reagent **10** (Scheme 2): Reagent **9** (202 mg) was dissolved in 2.5 mL dry DMF and 63 μL osmium tetroxide solution (2.5 wt % in *tert*-butanol) was added and stirred for five minutes. Then oxone (615 mg) was added and the solution was stirred for three hours, followed by the addition of excess Na_2_SO_3_. To extract the product, a suspension of DCM and HCl (1 M) was used five times, followed by brine and MgSO_4_, filtered and dried by rotary evaporator to obtain pure product **10**.^63^^1^H NMR (DMSO d_6_): δ = 8.71 (m, 4H), 8.08 (d, 2H), 8.00 (d, 2H), 7.91 (m, 2H), 7.83 (m, 4H), 7.56 (m, 3H) ppm. ^13^C NMR (DMSO d_6_): δ = 166.91, 132.50, 131.84, 129.89, 129.16, 128.20, 126.92, 122.33, 102.17, 95.35, 88.94 ppm. (FT-ICR) mass spectroscopy: *m*/*z* calcd. for C_31_H_18_O_2_ [M – H]^+^: 421.1223 found 421.1228.

**Scheme 3:**
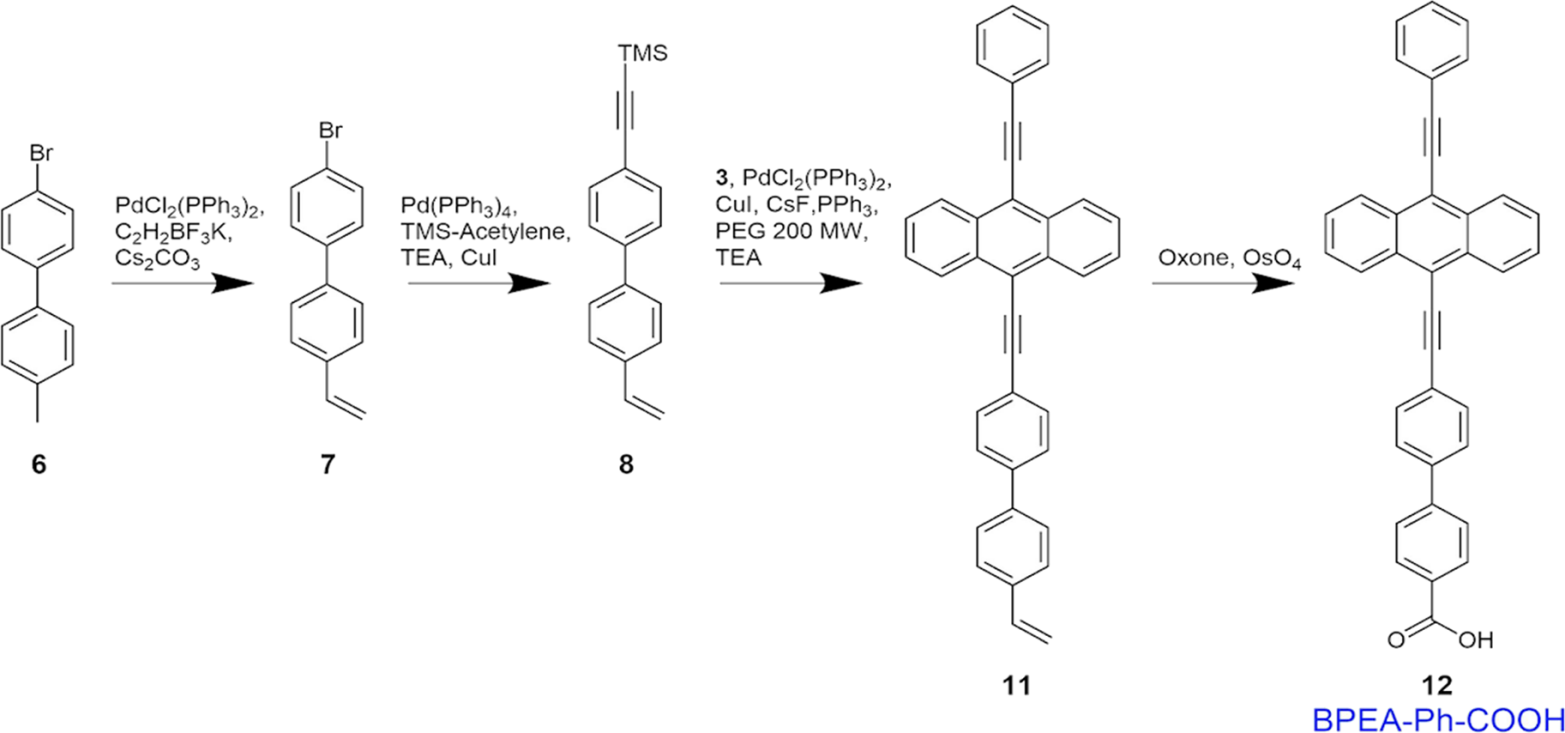
Schematic of the Synthesis of Reagent **12** (BPEA-Ph–COOH, Termed BPEA-Ph in the Results and Discussion)

Synthesis of reagent **7** (Scheme 3): 4-Bromo-4’-iodo-1,1’-biphenyl (reagent **6**, 1.0 g), potassium vinyltrifluoroborate (373.2 mg), cesium carbonate (36.3 mg), and bis(triphenylphosphine)palladium(II) dichloride (39.1 mg) were added and stirred in THF (5 mL) and deionized water (0.56 mL). The suspension was bubbled for 20 min and then refluxed at 78 °C for 24 h. The final crude suspension was evaporated and chromatographed on silica gel with hexane to obtain pure product **7**. ^1^H NMR (CDCl_3_): δ = 7.77 (d, 2H), 7.57 (d, 2H), 7.42 (dd, 4H), 6.76 (dd, 1H), 5.82 (d, 1H), 5.30 (d, 1H) ppm.

Synthesis of reagent **8** (Scheme 3): Reagent **7** (1139 mg), tetrakis(triphenylphosphine) palladium(0) (152.5 mg), and trimethylsilylacetylene (648 mg) were added to a mixture of toluene (19.7 mL) and triethylamine (8.4 mL). Then the suspension was bubbled with N_2_ for 20 min and copper(I) iodide (25 mg) was added. The suspension was stirred and refluxed at 55 °C for 24 h. The final crude was evaporated and chromatographed on silica gel with hexane to obtain pure product **8**. ^1^H NMR (CDCl_3_): δ = 7.53 (m, 8H), 6.76 (dd, 1H), 5.81 (d, 1H), 5.29 (d, 1H), 0.28 (s, 9H) ppm.

Synthesis of reagent **11** (Scheme 3): Reagent **3** (320.9 mg), copper(I) iodide (10.3 mg), cesium fluoride (273 mg), bis(triphenylphosphine)palladium(II) dichloride (18.9 mg), and triphenylphosphine (14.1 mg) were added to a 25 mL flask under N_2_. Then reagent **8** (372 mg), polyethylene glycol (0.32 mL), triethylamine (2.89 mL) and deionized water (0.16 mL) were injected into the 25 mL flask. The suspension was bubbled for 15 min and stirred at 80 °C for 16 h. The final crude was extracted and washed with deionized water three times, then brine and MgSO_4_, filtered and dried by rotary evaporator to obtain product **11**.^62^^1^H NMR (CDCl_3_): δ = 8.71 (m, 4H), 7.86 (d, 2H), 7.80 (d, 2H), 7.71 (d, 2H), 7.65 (m, 6H), 7.54 (d, 2H), 7.46 (m, 3H), 6.78 (dd, 1H), 5.85 (d, 1H), 5.31 (d, 1H) ppm.

Synthesis of reagent **12** (Scheme 3): Reagent **11** (213.6 mg) was dissolved in 2.24 mL dry DMF, and 56 μL osmium tetroxide solution (2.5 wt % in tert-butanol) was added and stirred for 5 min. Then oxone (546.4 mg) was added to the solution which stirred for 3 h followed by the addition of excess Na_2_SO_3_. To extract the product, a suspension of DCM and HCl (1 M) was used five times, followed by brine and MgSO_4_, filtered and dried by rotary evaporator to obtain pure product **12**.^63^^1^H NMR (DMSO d_6_): δ = 8.73 (m, 4H), 7.91 (d, 2H), 7.84 (d, 2H), 7.65 (d, 2H), 7.63–7.60 (m, 6H), 7.57 (d, 2H), 7.54 (m, 3H) ppm. ^13^C NMR (DMSO d_6_): δ = 133.22, 132.20, 132.05, 131.53, 128.81 ppm. (FT-ICR) mass spectroscopy: *m*/*z* calcd. for C_37_H_22_O_2_ [M – H]^+^: 497.1536, found 487.1548.

#### Synthesis of ZnO Quantum Dots

In a typical synthesis,^64^ 0.551 g of zinc acetate was measured into a 100 mL three-neck round-bottom flask with a stir rod. To this flask, 25 mL of anhydrous DMSO was added to dissolve the zinc acetate, while keeping the flask under inert atmosphere. In a separate vial, 0.771 g of solid TMAH and 7.7 mL of anhydrous ethyl alcohol were added and agitated until dissolution of the TMAH occurred. The TMAH in ethanol solution was added dropwise to the flask with the zinc acetate solution at a rate of 9 mL per hour. The solutions were allowed to react for various times (1 min, 90 min, and 6 h to obtain 3.1, 4.1, and 6.6 nm QDs) following the addition of the TMAH/ethanol solution. All aliquots were treated with the same cleaning procedure, described below.

Each synthesis solution was divided evenly between three 50 mL centrifuge tubes (roughly 10 mL aliquots) and precipitated via the addition of 14 mL of ethyl acetate. The solutions were then centrifuged for 10 min at 8700 rpm. The supernatant was decanted, leaving a white pellet of ZnO QDs. The pellet was resuspended in ethyl acetate and centrifuged once more. In a 50 mL centrifuge tube, 0.40 mL of oleic acid was mixed in 40 mL of toluene to prepare a 31.4 mM solution of oleic acid in toluene. This oleic acid solution (10 mL) was added to each pellet and sonicated until dissolution. The quantum dots were then reprecipitated from the toluene solution by the addition of 10 mL of methanol. The solutions were then centrifuged for 10 min at 8700 rpm. The supernatant was decanted, and the oleic acid – capped QDs were redispersed in 5 mL of DCM for storage. UV–vis absorption spectra were used to determine the quantum dot sizes,^66^ which tended to increase over time due to Ostwald ripening. UV–vis was also used to establish QD concentrations based an equation provided by Lommens et al.^89^ Due to the QDs growing in solution over time, samples were stored dried and in a −20 °C freezer to maintain size. Reported sizes are based on UV–vis spectra for optical transient absorption (∼5.8 nm), and on electron microscopy for EPR and steady-state optical spectroscopy (3.1, 4.1, and 6.6 nm).

### Preparation of Dye Molecule-ZnO Conjugates for Optical and EPR Studies

#### Transient Absorption Samples

To optimize transient absorption (TA) data collection, we prepared solutions with an optical density of ∼ 1 and a path length of 2 mm (for use with 2 mm cuvettes). Stock solutions were prepared in 3 mL THF using known extinction coefficients (Figure S2).^89^ Two solutions were prepared: one containing 21 μM BPEA and 10 μM ZnO QDs (5.8 nm diameter), while the other contained 22 μM BPEA-Ph and 29 μM ZnO QDs (5.8 nm diameter). The solutions were dried in an inert atmosphere and resuspended in 0.6 mL of toluene immediately prior to transient absorption experimentation. The resulting solutions had concentrations of 108 μM of BPEA with 51 μM ZnO QDs (solution 1) and 115 μM BPEA-Ph with 151 μM ZnO QDs (solution 2).

#### Steady State Absorption and Emission Experiments

Three solutions containing ∼ 2 μM of BPEA in 2.5 mL of THF were prepared in cuvettes corresponding to QD samples of varying sizes (3.1, 4.1, and 6.6 nm, respectively) using previously determined extinction coefficients of BPEA (Figure S2). Additionally, three solutions containing ∼ 1.6 μM of BPEA-Ph in 2.5 mL of THF were prepared in cuvettes following the same method (see Figures S3 and S4 for exact dye concentrations). UV–vis absorption and emission spectra of the six samples were recorded prior to adding varying amounts of their respective ZnO QD stock solution to each cuvette. After each addition we recorded the absorption and emission spectra. ZnO QDs were added to the solution to achieve roughly the following dye:QD ratios in THF: 40:1, 20:1 15:1, 7:1, 4:1, 2:1, and 1:1. The spectra containing detailed concentration information on ZnO QDs and dye are presented in Figures S3 and S4.

#### Preparation of Dye Molecule-ZnO Samples for EPR

For a typical EPR sample, we prepared solutions with an optical density of ∼ 5 given a path length of 2 mm, the approximate diameter of the EPR tubes. A solution containing ∼ 100 μM dye in 3 mL of THF was prepared in a cuvette using the known extinction coefficient of the dye molecule (see Figure S2 for extinction data). Then, various amounts of ZnO QDs (2.8, 4.0, and 5.3 nm) were added, resulting in concentrations ranging from 58 μM to 210 μM solution of ZnO QDs in the cuvette. Aliquots (50 μL) were taken from the cuvette and used to determine the ratio of dye molecules to ZnO QDs in solution. There was therefore an average of ∼ 1 dye molecules per ZnO QD in solution. The concentrated solution was dried in an inert atmosphere and stored. These ZnO QD – BPEA (-Ph) conjugates were resuspended in 0.1 mL of toluene prior to being transferred into EPR tubes. The optically determined concentrations of each EPR sample are summarized in Table S2.

### Instrumentation

#### Nuclear Magnetic Resonance

^1^H and ^13^C nuclear magnetic resonance spectra were collected using a Bruker AV 400 MHz spectrometer. Deuterated DMSO was used as the solvent for all samples. All spectra were recorded at room temperature. Chemical shifts are reported in δ(ppm). MestReNova software was used for integrations of the spectra.

#### Mass Spectrometry

Fourier-transform ion cyclotron resonance (FT-ICR) mass spectroscopy was performed on a Bruker SolariX. Mass spectrometry was performed on the BPEA and BPEA-Ph dye molecules. The various dyes were each dispersed in a methanol/chloroform solution for a total volume of 100 μL and concentration of ∼ 15 μM.

#### Transmission Electron Microscopy (TEM)

Dilute solutions in hexanes were added dropwise to the carbon side of the TEM grids (CF-400-Cu, Electron Microscopy Sciences), allowing solvent to evaporate after each drop. Samples were imaged at the University of Massachusetts, Amherst, Electron Microscopy core facility, where a FEI Tecnai T-12 Transmission Electron Microscope with 120 kV acceleration voltage was used to characterize the sample. The average diameters of the particles were obtained using Image-J (Figure S1).

#### Steady State Photoluminescence (PL)

Photoluminescence spectra were collected with a Horiba Scientific Quanta Master 8075–21-C using an excitation wavelength of 400 nm. In a typical measurement both the excitation and emission slits were kept at 2 nm and voltage bias was set to 5 V. The spectra were collected from 410 to 700 nm, with a step size of 1 nm and integration time of 0.1 s.

#### UV–Vis Absorption Measurements

Ultraviolet visible (UV–vis) absorption spectra were collected using an Agilent Technologies Cary 60 UV–vis spectrophotometer. The BPEA/BPEA-Ph – ZnO QD conjugates were dispersed in THF and measured in quartz cuvettes with 1 cm path lengths.

#### Femtosecond Transient Absorption Spectroscopy (fsTA)

An ultrafast transient absorption system with a tunable pump and white-light probe was used to measure charge dynamics of the dye - ZnO QD molecular system as a function of pump–probe delay time. The laser system consists of a regeneratively amplified Ti:sapphire oscillator (Coherent Libra), which delivers 4-mJ pulse energies centered at 800 nm with a 1-kHz repetition rate. The pulse duration of the amplified pulse is approximately 100 fs. The laser output is split by an optical wedge to produce the pump and probe beams and the pump beam wavelength is tuned using a Coherent OPerA optical parametric amplifier. The probe beam is focused onto a sapphire plate to generate a white-light continuum probe. The transient absorption spectra are collected with a commercial absorption spectrometer (Helios, Ultrafast Systems LLC). The temporal behavior is monitored by increasing the path length of the probe pulse and delaying it with respect to the pump pulse with a linear translation stage (minimum step size 16 fs). The pump wavelength was maintained at 420 nm with a pulse power between 100 nJ to 300 nJ. Residual pump light was filtered out of the collection optics using cross-polarization.

#### EPR Spectroscopy

For preparation of EPR samples, concentrated solutions of ZnO QD–BPEA (−Ph) conjugates with different sizes of ZnO QD (3.1, 4.1, and 6.6 nm), which had been dried and stored in an inert atmosphere, were dissolved in deoxygenated toluene inside a nitrogen atmosphere glovebox. The solution was then filled into 4 mm o.d. EPR quartz tubes and sealed in the glovebox. Subsequently, the samples were quickly frozen in liquid nitrogen and transferred into a precooled EPR resonator.

Continuous wave (cw) time-resolved EPR measurements were conducted at X-band (9.7 GHz) using an ELEXSYS E580 spectrometer that was equipped with a dielectric ring resonator (Bruker ER 4118X-MD5-W1). These measurements were carried out at a temperature of 20 K which was provided by liquid helium gas-flow cryostat (CF935, Oxford Instruments, UK) and an ITC temperature controller (Oxford Instruments, UK). Direct light excitation of the EPR samples in the resonator of the spectrometer was achieved using an OPO (basiScan, GWU Lasertechnik, Germany) pumped by an ns pulsed Laser (Nd:YAG Laser, INDI, Spectra-Physics/Newport, operating at 20 Hz). Light was delivered to the resonator through an optical fiber. Excitation wavelength was 450 nm and typical incident light intensities at the sample were ∼ 2 mJ per pulse. Direct detection TR-EPR (also referred to as transient EPR)^79,80^ experiments were performed in cw mode without field modulation and with a low (0.2 mW) microwave power at various applied external magnetic fields as a function of time. Experiments were performed with both a narrow field range (∼20 mT) to resolve the spin-correlated radical pair and with a wider field range (∼150 mT) to resolve the molecular triplet.

### Femtosecond Transient Absorption Data Analysis for BPEA and BPEA-Ph

Data sets were processed and analyzed using the Surface Xplorer Software (Ultrafast Systems) by subtracting background, and applying chirp correction. To analyze the BPEA* and BPEA^•+^ kinetics separately, a spectral deconvolution was performed on the transient spectra at each time point. The basis spectra for the excited state of the dyes (BPEA* and BPEA-Ph*) were assigned to the 5 ps time point. Whereas the basis spectra for the cation state of the dyes (BPEA^•+^ and BPEA-Ph^•+^) were assigned to the 6000 ps time point. These basis spectra were chosen due to their corresponding spectra having only one spectral signature, with the excited state signature at ∼ 580 nm and the cation signature at ∼ 700 nm.^69^ The ZnO radical anion is not expected to appear in the visible region. It typically presents as a broad absorption in the IR.^42,90^ Once spectral reconvolution was performed on the transient spectra at each time point, comparisons between the data and spectral reconvolution could be made (Figure S7). Overall, there was good agreement between the experimental and reconvolution spectra. Results from the spectral deconvolution are shown in the main text, highlighting relative populations of BPEA* and BPEA^•+^ (Figure 3). The data was normalized to the BPEA* relative population. Kinetic fits using eqs 2 and 3 were applied to the cation and excited state, respectively. Parameters from these fits include normalized amplitudes (*a*_*i*_) and time constants (τ_*i*_) shown in Table S4.
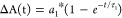
2
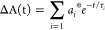
3

### EPR Data Processing and Modeling

#### TR-EPR SCRP Data Processing

The TR-EPR data with a narrow field sweep (∼20 mT) were used. Each experiment provides a two-dimensional data set of microwave absorption/emission as a function of magnetic field and time (Figures S8–S9). Spectra were processed by first subtracting the prelaser signal at each magnetic field (dark background), and then subtracting a field-dependent linear background from each point in time (to remove the triplet and other laser-induced background signals). Transient spectra were then integrated between 600 – 1500 ns after laser excitation for all samples. A minor improvement in the correspondence between simulations and data was obtained when longer-lived transient spectra (3000 – 6000 ns) were integrated and subtracted from the early time spectra (600 – 1500 ns). We hypothesize that these longer-lived transients are associated with sample degradation since they were most prominent in samples that decomposed more rapidly. The resulting SCRP spectra were normalized to the maximum signal intensity and exhibit the expected *e, a, e, a* polarization pattern.

#### Spin-Correlated Radical Pair Simulation Parameters and Details

The SCRP data processing and simulations were performed using Matlab R2023a (MathWorks Inc., Natick) and the EasySpin toolbox (version 6.0.4).^84,85^ Four transitions are expected with peak positions determined by *J* (exchange coupling, isotropic), *D* (dipolar coupling, anisotropic), and the *g* values of the spins (*g*_*A*_ and *g*_*B*_). The *g*-anisotropy and hyperfine interactions are too small to be resolved (see results of DFT calculations, Table S9, S11). Note that conventions vary on the sign and magnitude of *J* and *D*, and we have chosen to use the convention employed by EasySpin with the following Hamiltonian:
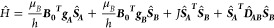
4

The positions of the four transitions for one orientation of the radical pair with respect to the external field (defined by θ) is then described by Figure S10b, and eqs 5 – 8. Averaging over all orientations produces broadened peaks (Figure S10c).
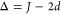
5
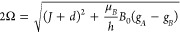
6
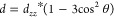
7
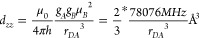
8

The two-spin system was defined in EasySpin (Sys.S = [1/2 1/2]) with the following adjustable parameters: *J* (Sys.J), *d*_*zz*_ (Sys.dip), *g* values of ZnO and BPEA (Sys.g), inhomogeneous broadening implemented with the *g* strain (Sys.gstrain), and lifetime (Lorentzian) broadening (Sys.lwpp). Note that the use of two broadening schemes was necessary to simulate different broadening for the different radicals. The relative populations of T_+_, T_0_, T_–_, and S were varied (Sys.initState) with the ‘coupled’ option. Additionally, the ZnO peaks were manually adjusted by factors of 0.5 – 0.7 relative to the BPEA peaks (termed “ZnO weight” in Table S4). We hypothesize that this adjustment is required owing to the fast spin relaxation on ZnO. In order to maintain physically meaningful parameters, we placed a few constraints on the parameter space. The dipolar coupling was calculated based on radical pair separation (r_DA_) in accordance with eq 8. The exchange coupling was set to −1.0 MHz for the shortest separation (BPEA – 3.1 nm, r_DA_ = 2.5 nm) and allowed to decay exponentially in accordance with *J* ∝ *e*^–β(*r* – *r*_0_)^ with β = 0.46 Å^–1^, which is in line with values reported for oligophenyls.^86^ We note that reasonable simulations were also produced when this closest exchange coupling was anywhere in the range of −0.1 to −10 MHz. These values are well within the expected range for reported radical pairs with similar physical separation.^35,36^ Other parameters were adjusted to improve agreement between experimental and simulated SCRP spectra. Notably, we found that the BPEA(-Ph)^•+^*g*-value was consistent across all samples at 2.0028, which is consistent with electronic structure calculations (see Table S9, S11). Furthermore, the *g*-strain was 0.0025 for all BPEA(-Ph)^•+^ peaks, which corresponds to an effective line broadening of 12 MHz, on the order of the largest hyperfine couplings predicted from electronic structure calculations (Table S9, S11).

#### TR-EPR Triplet Data Processing

The TR-EPR data with a wide field sweep (∼150 mT) were used. Each experiment provides a two-dimensional data set of microwave absorption/emission as a function of magnetic field values and time. Spectra were processed by first subtracting the prelaser signal at each magnetic field, and then subtracting a field-dependent linear background from each point in time (to remove laser-induced background signals). Transient spectra were then integrated between 400 – 1000 ns after laser excitation for all samples. The resulting spectra were normalized to the maximum signal intensity (excluding the sharp SCRP resonances). We would like to mention that the TR-EPR method is not well suited to study spin dynamics since the continuous microwave irradiation influences the kinetics. For this reason, we do not discuss the spin dynamics in the manuscript. That would be better investigated with pulsed EPR methods, which is not part of this manuscript and would require additional experiments.

#### Triplet Simulation Parameters and Details

Triplet simulations were performed using Matlab R2023a (MathWorks Inc., Natick) and the EasySpin toolbox (version 6.0.4).^84,85^ The spin system was defined in EasySpin (Sys.S = 1) with the following adjustable parameters: the *g* value of the triplet (Sys.g), the zero-field splitting (ZFS) parameters D and E (Sys.D = [D, E]), and anisotropic line broadening (Sys.HStrain = [H_*x*_ H_*y*_ H_*z*_]). Two mechanisms for triplet formation were considered: RP-ISC and SO-ISC (see main text). For RP-ISC the initial population was set to T_0_ (Sys.initState = {[0 1 0], ‘eigen’}), and for SO-ISC initial populations were defined in zero field x, y, and z bases (Sys.initState = {[Tx Ty Tz], ‘xyz’}). The relative contributions of these two mechanisms as well as the ZFS and line broadening parameters were adjusted to improve correspondence between simulated and experimental spectra. We find that BPEA triplets could be well described by the RP-ISC mechanism, while the BPEA-Ph simulations required contributions from both mechanisms. We also note that the ZFS parameters for BPEA-Ph and BPEA are quite similar, highlighting the similarity of these chromophores. In contrast, the ZFS parameter D for SO-ISC in BPEA-Ph was slightly larger, indicating a change in its surrounding. We attribute this triplet to BPEA-Ph molecules not bound to the ZnO QDs.

## Supporting Information Available

Information on sample concentrations used in TA and EPR measurements, electron microscopy analysis, extinction coefficient calculations, additional absorption and emission spectra of QD – molecule conjugates with Stern–Volmer analysis, photoluminescence lifetime measurements of QD – molecule conjugates, radical pair energy calculations, transient absorption fitting parameters, full two-dimensional TR-EPR data sets, radical pair and triplet simulation parameters, electronic structure calculations on BPEA and BPEA-Ph (PDF)
