# Supplementary material for: Tunable Spin Qubit Pairs in Quantum Dot–Molecule Conjugates
Source: ACS Nano. 2025 Mar 19;19(12):12194–207. doi: 10.1021/acsnano.5c00288 (PMC11966761; doi:10.1021/acsnano.5c00288)
Supplement: Supplementary file 1 — nn5c00288_si_001.pdf [file nn5c00288_si_001.pdf]

Supporting Information for:

Tunable spin qubit pairs in quantum dot – molecule conjugates

Autumn Y. Lee,[1,⊥] Mandefro Teferi,[2,⊥] Frida S. Hernandez,[1] Amisha Jain,[1] Tiffany Tran,[3] Kefu Wang,[3] Tomoyasu Mani,[5] Adam M. Schwartzberg,[4] Ming Lee Tang,[3] Jens Niklas,[2] Oleg G. Poluektov,[2] Jacob H. Olshansky[1,*]

[1]Department of Chemistry, Amherst College, Amherst, Massachusetts 01002, United States
[2]Chemical Sciences and Engineering Division, Argonne National Laboratory, Lemont, Illinois 60439, United States
[3]Department of Chemistry, University of Utah, Salt Lake City, Utah 84112, United States
[4]The Molecular Foundry, Lawrence Berkeley National Laboratory, Berkeley, CA 94720, United States
[5]Department of Chemistry, University of Connecticut, Storrs, Connecticut 06269-3060, United States
[⊥]These authors contributed equally to this study.

Email: [*]jolshansky@amherst.edu

Contents

Concentrations of samples for TA and EPR 3
Electron Microscopy Analysis 4
Additional Optical Experiments 5
Dye molecule extinction coefficients 5
Absorption and emission of BPEA (BPEA-Ph) with the addition of ZnO QDs 6
Stern-Volmer Quenching of BPEA (BPEA-Ph) with ZnO QDs 8
Photoluminescence lifetime measurements of BPEA and BPEA-Ph 10
Radical Pair Energy Calculations 11
Transient absorption fitting parameters 12
EPR experiments and simulations 13
TR-EPR SCRP Full Data Sets 13
TR-EPR SCRP Simulation parameters 15
Analysis of *g* strain based on ZnO size inhomogeneity 17
TR-EPR Triplet Full Data Sets 18
TR-EPR Triplet Simulation Parameters 19
BPEA and BPEA-Ph Electronic Structure Calculations 21
BPEA – Radical Cation 22
BPEA - Neutral Triplet 24
BPEA-Ph – Radical Cation 26
BPEA-Ph – Neutral Triplet 28
References 29

Concentrations of samples for TA and EPR

Table S1. Concentration calculations of each TA sample using optical measurements. They include moles of dye molecule and ZnO QDs in solution, volume used to resuspend the dye molecule – ZnO QD samples for TA measurements, and the concentration of dye molecule and ZnO QDs in solution for the TA measurement.

Sample	Moles of Dye	Moles of ZnO QDs	Volume of TA Sample (mL)	Concentration of Dye in TA Sample (µM)	Concentration of ZnO QDs in TA Sample (µM)	Molecules per ZnO QD
BPEA – ZnO (5.8 nm)	6.48E-08	3.05E-08	0. 6	108.1	50.8	2.1
BPEA-Ph – ZnO (5.8 nm)	6.88E-08	9.08E-08	0. 6	114.7	151.4	0.8

Table S2. Concentration calculations of each EPR sample using optical measurements. They include concentration of dye molecule and ZnO QDs in solution before they are dried for ease of transport, volume used to resuspend the dye molecule – ZnO QD samples for EPR measurements, and the concentration of dye molecule and ZnO QDs in solution for each EPR measurement.

Sample	µM Dye (cuvette)	µM ZnO (cuvette)	Volume in EPR tube (mL)	µM Dye (EPR tube)	µM ZnO QDs (EPR tube)	Dye per ZnO QD Ratio
BPEA – ZnO (3.1 nm)	80	62	0. 1	2500	1900	1.3
BPEA-Ph – ZnO (3.1 nm)	85	200	0. 1	2600	6200	0.4
BPEA – ZnO (4.1 nm)	97	61	0. 1	3000	1900	1.6
BPEA-Ph – ZnO (4.1 nm)	130	210	0. 1	4200	6500	0.6
BPEA – ZnO (6.6 nm)	98	60	0. 1	3100	1800	1.7
BPEA-Ph – ZnO (6.6 nm)	100	190	0. 1	3200	5900	0.5

Electron Microscopy Analysis

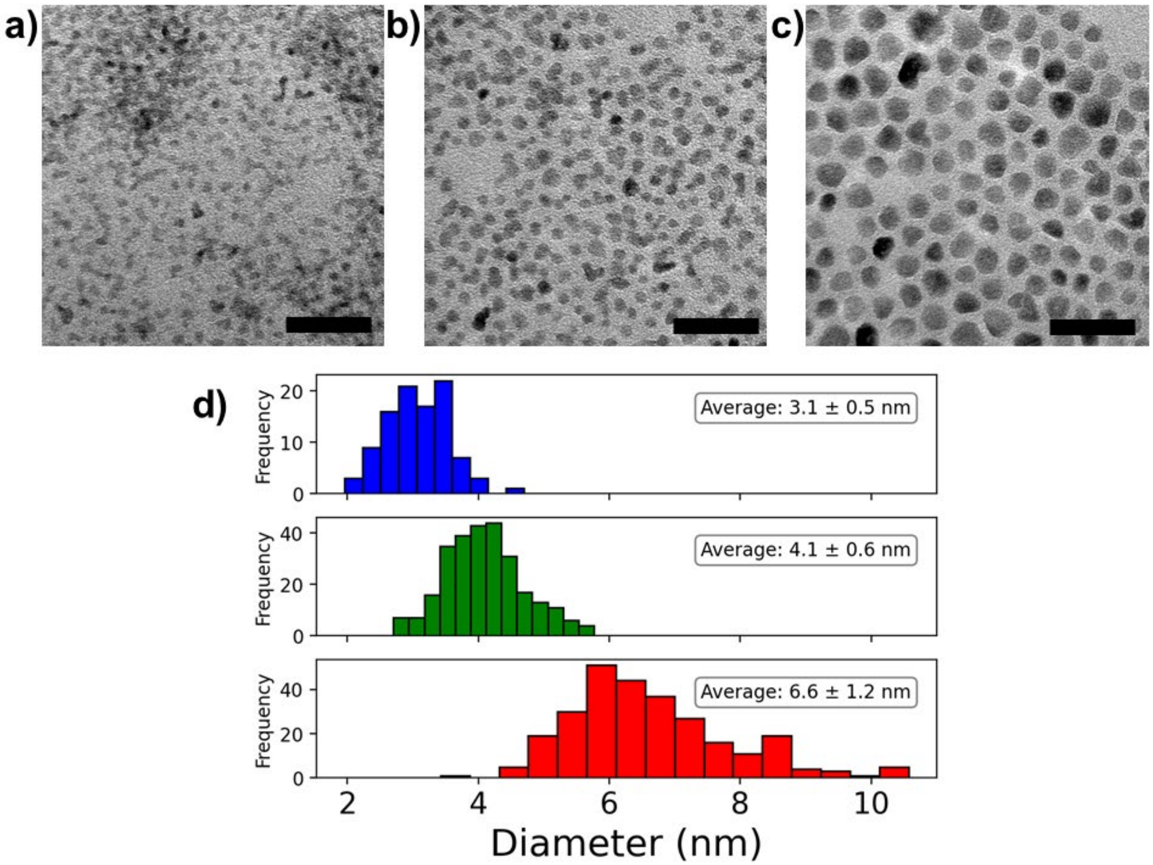

Figure S1. Transmission electron microscopy (TEM) images of 3.1 (a), 4.1 (b), and 6.6 (c) nm ZnO QDs (denoted by their mean size). Scale bar is 30 nm. d) Sizing histograms based on n = 100 individual measurements for 3.1 nm QDs and n = 273 individual measurements for the 4.1 and 6.6 nm QDs.

Additional Optical Experiments

Dye molecule extinction coefficients

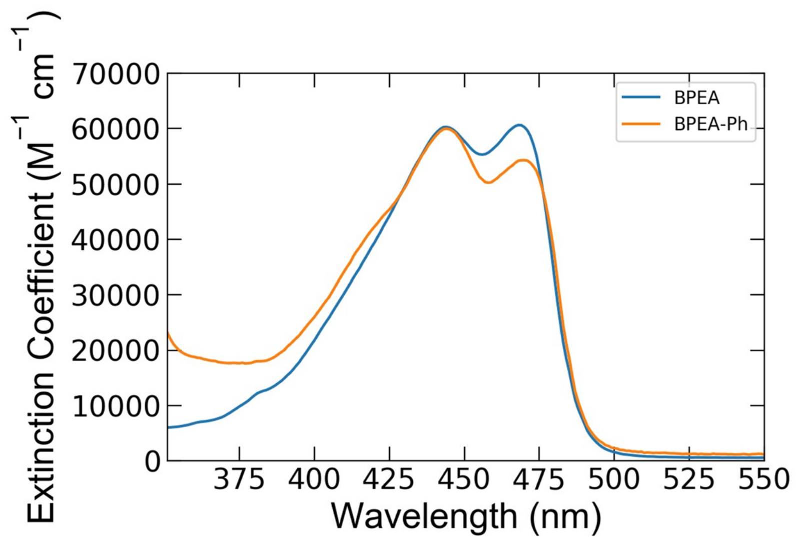

Figure S2. Extinction coefficients of BPEA and BPEA-Ph at various wavelengths. Notable wavelengths include 445 nm and 470 nm, the corresponding extinction coefficients for BPEA are 60,200 M^{-1} cm^{-1} and 60,900 M^{-1} cm^{-1} and for BPEA-Ph 59,900 M^{-1} $cm^{-1}$and 54,300 M^{-1} cm^{-1}, respectively.

Extinction coefficients for BPEA and BPEA-Ph were obtained using quantitative NMR (qNMR) with 600 µL of DMSO-d_6 and 5.0 µL of DCM as a standard. Following qNMR, three dilutions of each dye molecule NMR sample in 2.5 mL of THF were prepared. These were 500x, 250x and 125x dilutions from the NMR samples from which UV-Vis measurements were taken. The number of DCM molecules per dye molecule in the qNMR solution was determined by integrating known peaks of BPEA and BPEA-Ph and then comparing it to the DCM peak. Then the molarity of the dyes in UV-Vis solution was calculated. Finally, we employed the Beer-Lambert law to determine the extinction coefficient of BPEA and BPEA-Ph ranging from 350 nm to 550 nm.

Absorption and emission of BPEA (BPEA-Ph) with the addition of ZnO QDs

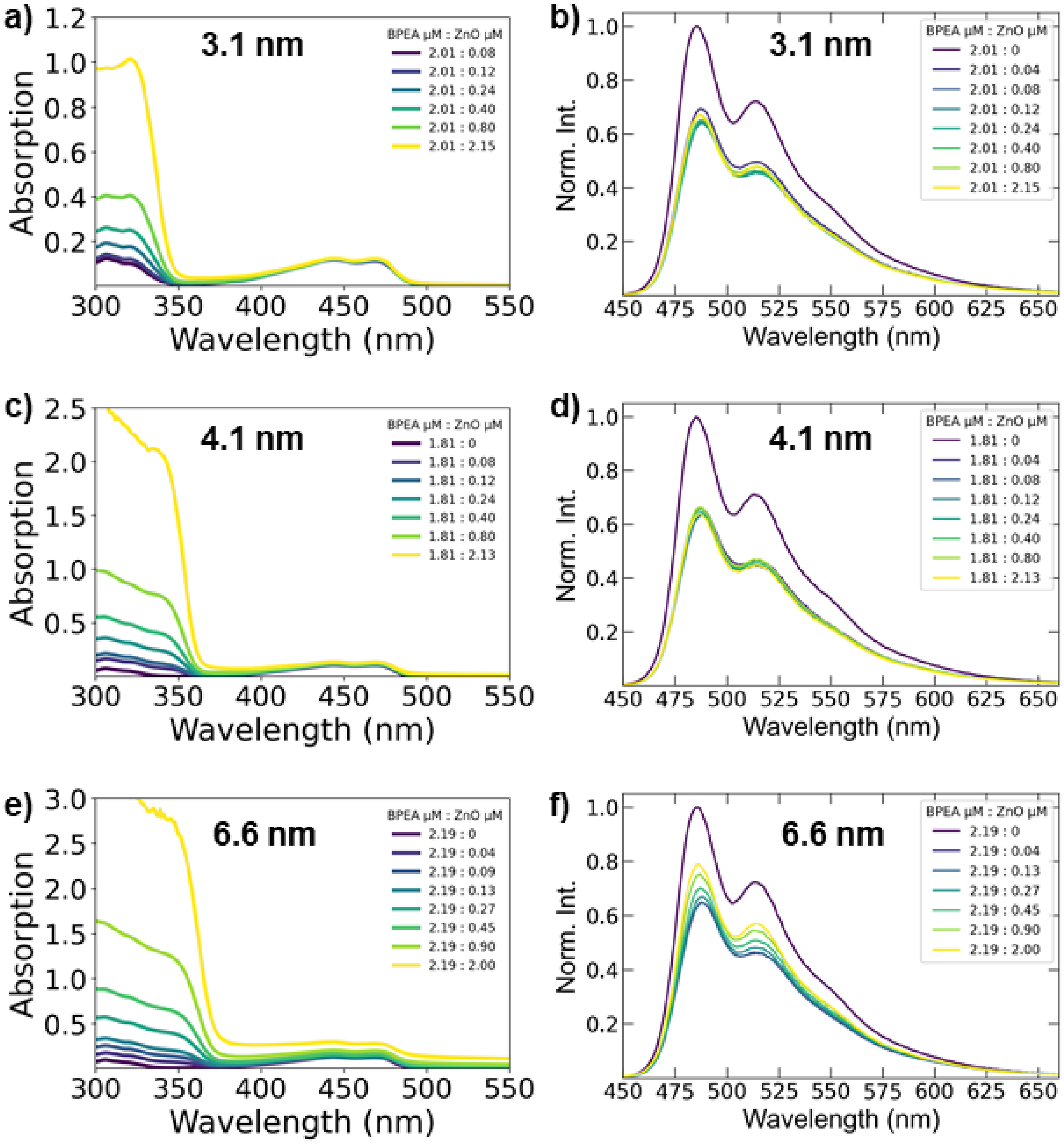

Figure S3. Steady state optical spectroscopy of dye – ZnO QD conjugates. Absorption (a, c, e) and emission spectra (b, d, f) of BPEA as ZnO QDs (3.1, 4.1, and 6.6 nm) are added to solution. All experiments were performed in tetrahydrofuran. Data with ~2 : 1 BPEA : ZnO are reproduced in Figure 2 of the main text.

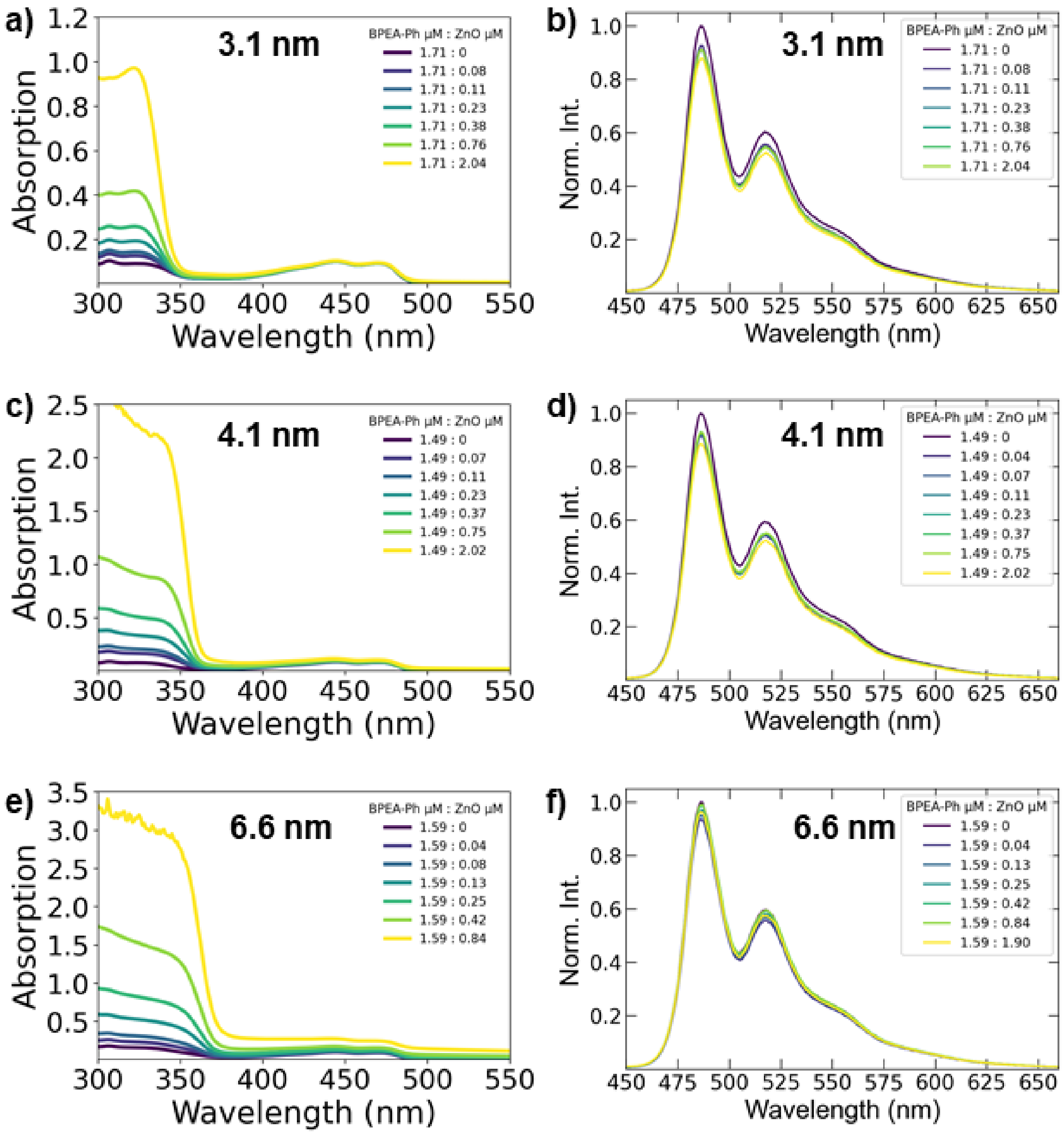

Figure S4. Steady state optical spectroscopy of dye – ZnO QD conjugates. Absorption (a, c, e) and emission spectra (b, d, f) of BPEA-Ph as ZnO QDs (3.1, 4.1, and 6.6 nm) are added to solution. All experiments were performed in tetrahydrofuran. Data with ~2 : 1 BPEA : ZnO are reproduced in Figure 2 of the main text.

Stern-Volmer Quenching of BPEA (BPEA-Ph) with ZnO QDs

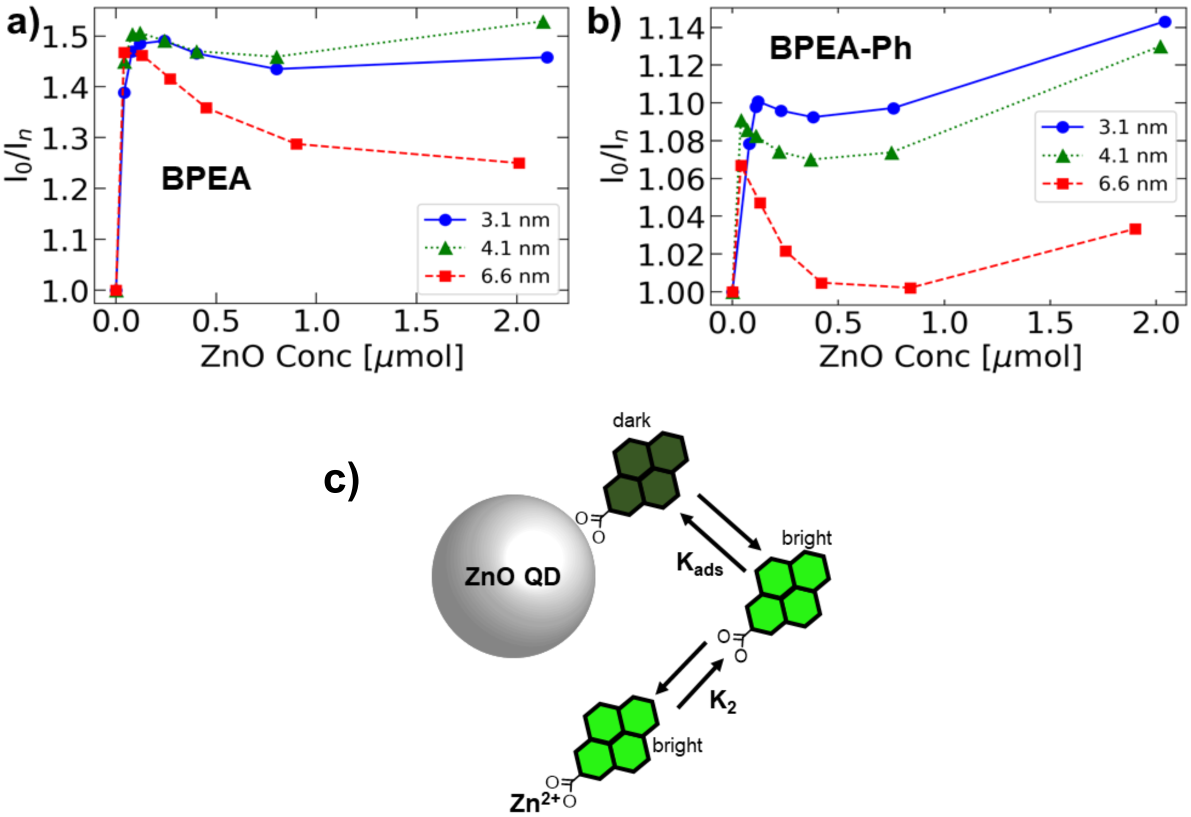

Figure S5. Stern-Volmer analysis based on quenching data in Figures S3 and S4. I_0 represents the intensity of BPEA (BPEA-Ph) emission without ZnO QDs, and I_n is the intensity of BPEA (BPEA-Ph) with varying ZnO concentrations. The a) BPEA solutions were ~1.8 – 2.2 μM while the b) BPEA-Ph solutions were ~1.5 – 1.7 μM. All experiments were performed in tetrahydrofuran at room temperature . c) Cartoon of proposed equilibrium mechanism that can explain the observed Stern-Volmer behavior.

BPEA fluorescence is quenched by ~33% upon addition of ZnO QDs of all three sizes, while the BPEA-Ph is only quenched by 5 – 10%. The Stern-Volmer behavior in all cases is highly abnormal since addition of quencher (ZnO) increases emission at higher concentrations. As discussed in the main text, this can be explained by a model in which free Zn^{2+} in the ZnO QD solutions competitively binds to BPEA (BPEA-Ph) (Figure S5c). The prior work that put forth this model observes very similar Stern-Volmer plots.[1–3] This explanation is further justified by the fact that we observed increased luminescence after adding zinc salts to QD – molecule conjugate solutions. We also attempted to isolate the QD – molecule conjugates with no free molecules by anti-solvent induced precipitation. The supernatant did indeed contain free dye molecules, which could be

removed. However, when the pellet was resuspended, additional free dye molecules were inferred from the fluorescence data. Presumably, the dynamic equilibrium described in Figure S5c drives additional molecules from the surface of the QDs. Additional centrifugation steps to remove free dye molecules inevitably resulted in the continued removal of all dye molecules from the QD surface. We should also note that photoluminescence lifetime experiments on QD – molecule conjugates detected only monoexponential radiative decay (2-3 ns) from the free BPEA or BPEA-Ph. This observation suggests that the equilibrium is somewhat slower than the radiative lifetime.

Photoluminescence lifetime measurements of BPEA and BPEA-Ph

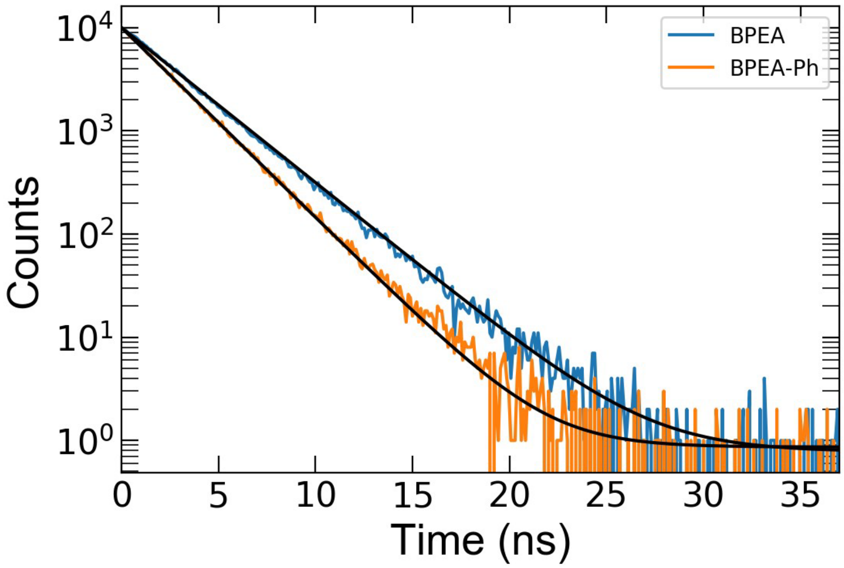

Figure S6. Fluorescence lifetime spectroscopy of BPEA (1.5 μM) and BPEA-Ph (0.53 μM) in tetrahydrofuran. Samples were photoexcited at 405 nm and emission detected at 450 nm. Mono-exponential fits were used to obtain the fluorescence lifetimes of the dye molecules: BPEA (2.9 ns) and BPEA-Ph (2.4 ns).

The lifetimes were measured using a Horiba Scientific DeltaFlex Modular Fluorescence Lifetime System and a DeltaDiode laser with a peak wavelength at 402 nm as an excitation source. The measurements were taken in a 400 ns time range at the wavelength corresponding to the maximum fluorescence intensity of the sample. The lifetime data (Figure S6) was fit to a mono-exponential function,

$$I(t) = B * e^{\left(-\frac{t}{\tau}\right)} + c, \quad \text{S1}$$

where B is the pre-exponential factor, t is time, τ is fluorescence lifetime, and c is a coefficient. We found that BPEA and BPEA-Ph have fluorescence lifetimes of 2.9 and 2.4 ns, respectively. The lifetimes were measured using the same samples utilized in steady state absorption and emission measurements, BPEA (1.5 μM) and BPEA-Ph (0.53 μM).

Radical Pair Energy Calculations

The energetics of the $BPEA^{\bullet +}$($-Ph^{\bullet +}$) – $ZnO^{\bullet -}$ QD radical ion pairs utilized in Figure 1 of the main text were calculated using the Weller expression:

$$\Delta G_{IP} = E_{ox} - E_{red} - \frac{e^2}{r_{DA} * \varepsilon_s} + e^2 \left(\frac{1}{2r_D} + \frac{1}{2r_A}\right)\left(\frac{1}{\varepsilon_s} + \frac{1}{\varepsilon_{sp}}\right) \qquad \text{S2}$$

where E_{ox} and E_{red} are the oxidation and reduction potentials of the donor (BPEA) and acceptor (ZnO QD) in a polar solvent (ε_{sp}), r_D and r_A are the radii of the donor and acceptor, r_{DA} is the donor-acceptor distance, e is the charge of the electron, and ε_s is the dielectric constant of the solvent that is used when spectroscopy is performed (ε_s = 2.38 for toluene).[4] The oxidation potential of BPEA was previously determined to be +1.21 V vs. SCE.[5] The reduction potentials of ZnO were calculated based on a bulk oxidation potential of -0.55 V vs. SCE,[6] and adjusted for the QDs based on experimental bandgap changes and assuming that 64% of this change occurs in the conduction band (this percentage is derived from the ratio of hole and electron effective masses). The radius of both electron donors (r_D) were approximated to be 0.7 nm based on the spatial extent of the anthracene moiety, and the radius of the acceptor (r_A) was approximated as the radius of the QD. Radical pair distances, found in the table below, were approximated as the distance between the middle of the anthracene moiety and the center of the ZnO QD. The dielectric constant of the polar solvent (ε_{sp}) used to determine the oxidative potential was 8.93 for DCM.

Table S3. Weller approximation calculations for BPEA and BPEA-Ph attached to various ZnO QD diameters.

**Dye – ZnO**	**E_{ox} (V vs SCE)**	**E_{red} (V vs SCE)**	**r_{DA} (nm)**	**ΔG_{IP} (eV)**
BPEA – ZnO QD (3.1 nm)	1.21	-0.75	2.5	2.19
BPEA-Ph – ZnO QD (3.1 nm)	1.21	-0.75	2.9	2.22
BPEA – ZnO QD (4.1 nm)	1.21	-0.62	3.0	2.06
BPEA-Ph – ZnO QD (4.1 nm)	1.21	-0.62	3.4	2.09
BPEA – ZnO QD (6.6 nm)	1.21	-0.57	4.3	2.02
BPEA-Ph – ZnO QD (6.6 nm)	1.21	-0.57	4.7	2.04

Transient absorption fitting parameters

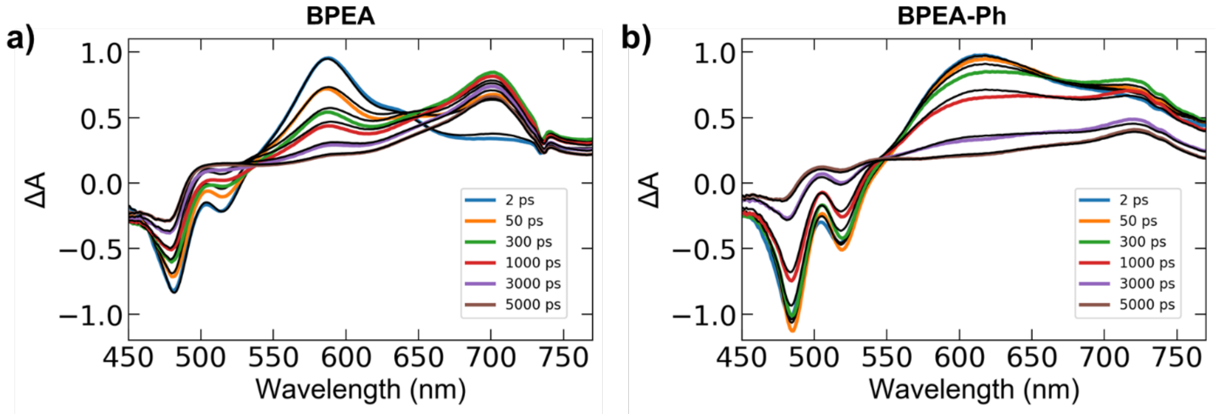

Figure S7. Comparison between experimental and simulated spectra based on a linear combination of basis spectra. a) Comparison for BPEA – ZnO QDs (5.8 nm) with experimental (color) and simulated (black) spectra at six time points. b) Comparison for BPEA-Ph – ZnO QDs (5.8 nm) with experimental (color) and simulated (black) spectra at six time points.

Table S4. Time constants (τ_i) and normalized amplitudes (a_i) for BPEA(-Ph)$^{\bullet+}$ (rise, Equation S3) and BPEA(-Ph)* (decay, Equation S4) in dye-ZnO QD (5.8 nm) conjugates.

**Electronic State**	**a_1**	**τ_1 (ps)**	**a_2**	**τ_2 (ps)**
**BPEA$^{\bullet+}$**	0.55	59	0	0
**BPEA***	0.53	54	0.44	2080
**BPEA-Ph$^{\bullet+}$**	0.21	410	0	0
**BPEA-Ph***	0.95	2010	0	0

EPR experiments and simulations

TR-EPR SCRP Full Data Sets

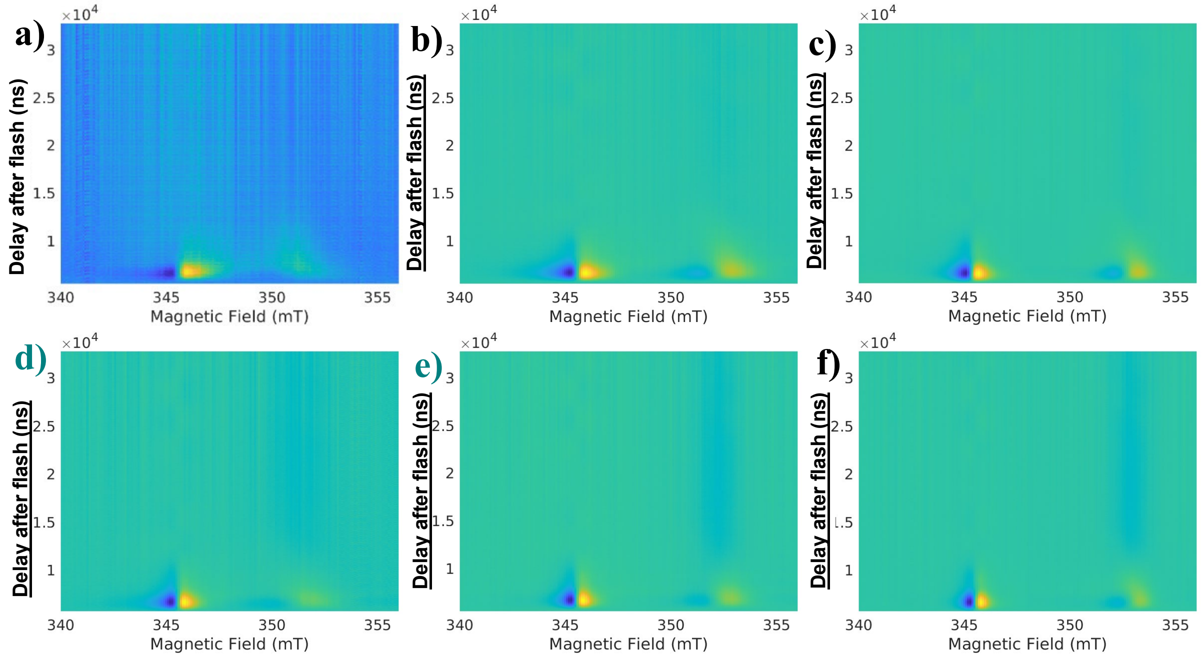

Figure S8. 2D contour plots of the SCRP region for BPEA with a) 3.1, b) 4.1, and c) 6.6 nm ZnO QDs and BPEA-Ph with d) 3.1 e) 4.1, and f) 6.6 nm ZnO QDs. Blue signifies emissive features and yellow signifies absorptive features of the SCRP.

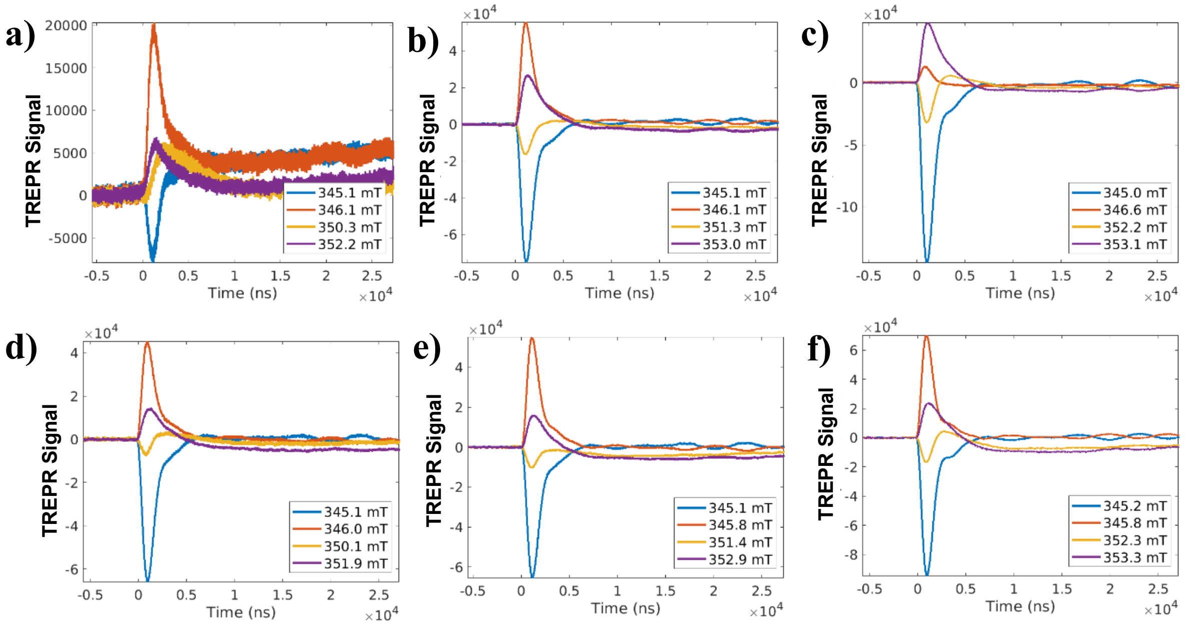

Figure S9. TR-EPR signal at field points corresponding to the *eaea* signal of the SCRP plotted over time for BPEA with a) 3.1, b) 4.1, and c) 6.6 nm ZnO QDs and BPEA-Ph with d) 3.1, e) 4.1, and f) 6.6 nm ZnO QDs. The oscillatory behavior can be ascribed to Rabi oscillations from continuous microwave irradiation, which also accelerates spin decoherence.

TR-EPR SCRP Simulation parameters

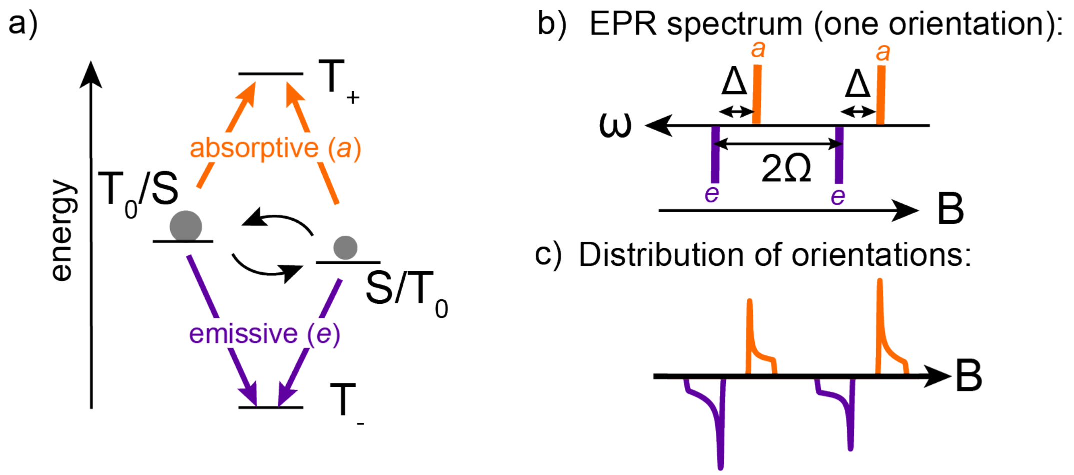

Figure S10. Overview of the radical pair model in presence of a strong magnetic field. a) Energy level diagram of the four spin states that compose the radical pair. Initially, only the $m_s = 0$ states are populated. b) Predicted stick diagram of the four transitions: two emissive (purple) and two absorptive (orange). c) A distribution of radical pair orientations will produce a distribution of dipolar couplings, which will broaden the four transitions. The unresolved hyperfine interactions and g-tensor anisotropy have been omitted for simplicity.

Table S5. BPEA-ZnO QD SCRP simulation parameters.

Parameters	d_{zz} (MHz)	J (MHz)	ZnO *g* value	ZnO gstrain	ZnO weight	Lorentzian linewidth (MHz) / est. T_2 (ns)	S / T_0 population
**BPEA – ZnO (3.1 nm)**	3.33	-1.0	1.972	0.014	0.5	28.0 / 36	1.0 / 0.0
**BPEA-Ph – ZnO (3.1 nm)**	2.13	-0.02	1.972	0.012	0.8	14.0 / 71	0.97 / 0.03
**BPEA – ZnO (4.1 nm)**	1.928	$-1.3*10^{-1}$	1.965	0.008	0.7	19.1 / 52	0.96 / 0.04
**BPEA-Ph – ZnO (4.1 nm)**	1.324	$-2.7*10^{-3}$	1.966	0.0085	0.8	11.2 / 89	0.95/ 0.05
**BPEA – ZnO (6.6 nm)**	0.655	$-7.5*10^{-4}$	1.961	0.0055	0.6	12.9 / 78	0.97/ 0.03
**BPEA-Ph – ZnO (6.6 nm)**	0.501	$-1.5*10^{-4}$	1.961	0.0055	0.6	8.4 / 119	0.96/ 0.04

Analysis of *g* strain based on ZnO size inhomogeneity

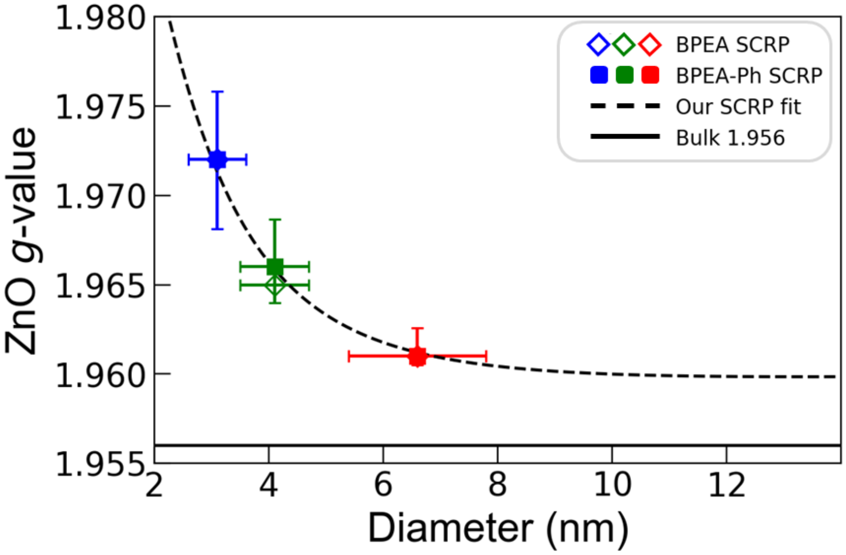

Figure S11. ZnO *g* value as a function of ZnO QD diameter. Experimental size distributions are shown as x-error bars and predicted *g* distributions are shown as y-error bars. This data is also shown in Figure 5a of the main text.

Table S6. Summary of linewidth analysis on ZnO EPR resonances, comparing predicted *g* distribution based on size distribution and observed *g* distribution from EPR simulations.

Sample	QD diameter (nm)	FWHM of size distribution based on TEM (nm)	FWHM of predicted *g* distribution based on size distribution and Equation 1	*g* strain (FWHM from SCRP simulations)
BPEA – ZnO	3.1	1.2	0.0091	0.012
BPEA-Ph - ZnO	3.1	1.2	0.0091	0.012
BPEA - ZnO	4.1	1.4	0.0055	0.008
BPEA-Ph - ZnO	4.1	1.4	0.0055	0.008
BPEA - ZnO	6.6	2.8	0.0024	0.0055
BPEA-Ph - ZnO	6.6	2.8	0.0024	0.0055

TR-EPR Triplet Full Data Sets

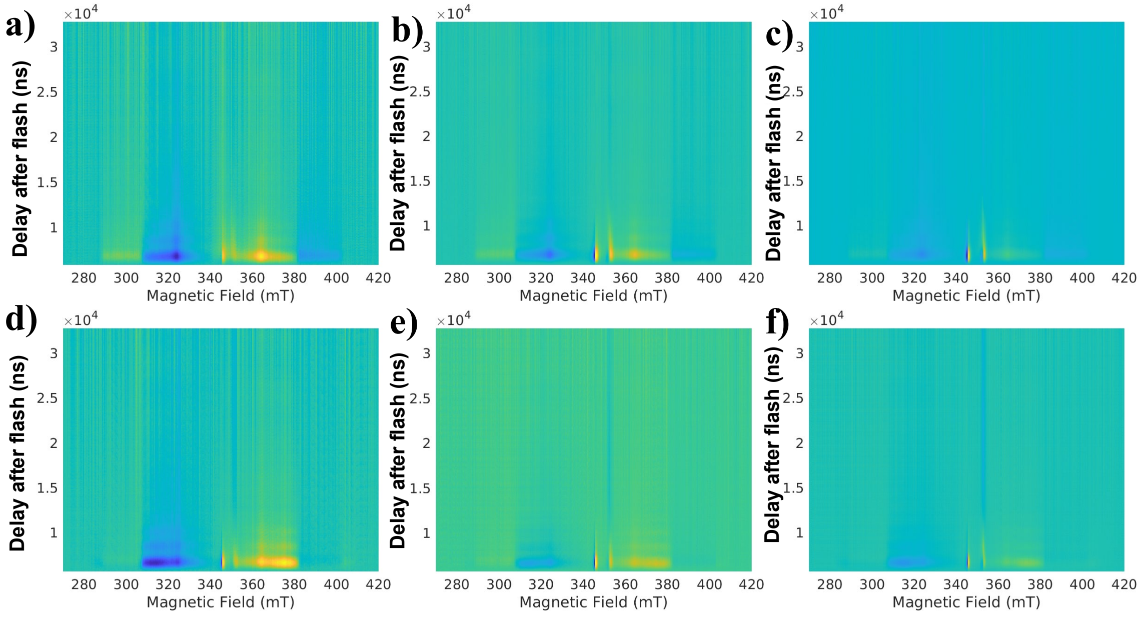

Figure S12. 2D contour plots of the triplet region for BPEA with a) 3.1, b) 4.1, and c) 6.6 nm ZnO QDs and BPEA-Ph with d) 3.1, e) 4.1, and f) 6.6 nm ZnO QDs. Blue signifies emissive features and yellow signifies absorptive features.

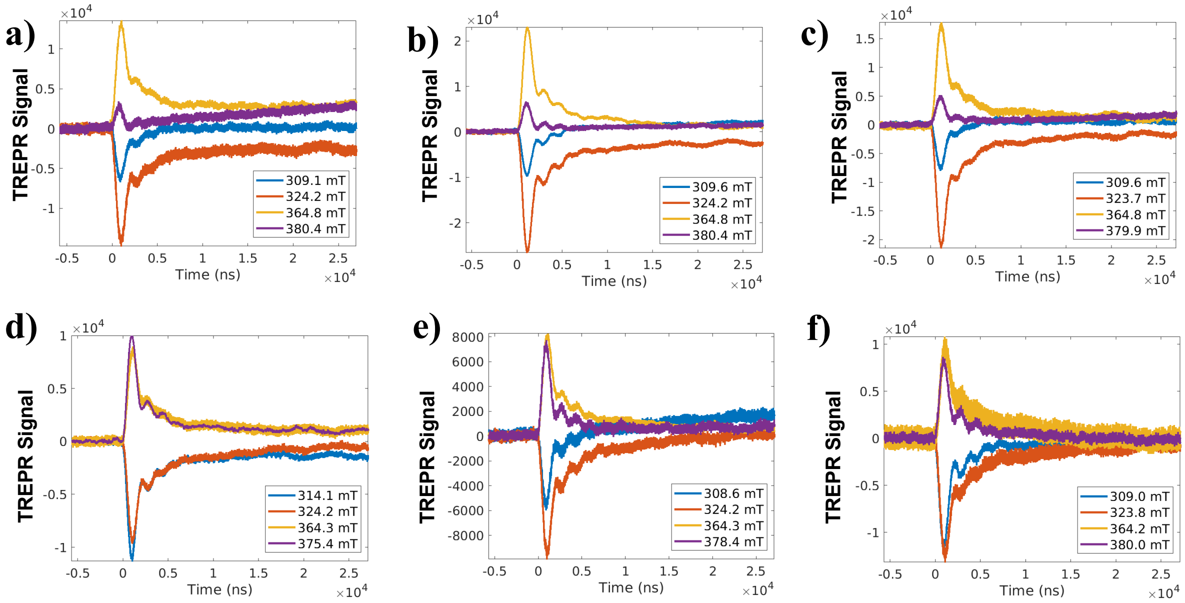

Figure S13. TR-EPR signal at field points corresponding to features of the triplet region plotted over time for BPEA with a) 3.1, b) 4.1, and c) 6.6 nm ZnO QDs and BPEA-Ph with d) 3.1, e) 4.1, and f) 6.6 nm ZnO QDs. The oscillatory behavior can be ascribed to Rabi oscillations from continuous microwave irradiation, which also accelerates spin decoherence.

TR-EPR Triplet Simulation Parameters

Table S7. BPEA triplet simulation parameters

Parameters	BPEA – ZnO (3.1 nm)	BPEA – ZnO (4.1 nm)	BPEA – ZnO (6.6) nm)
|D| (MHz)	1589	1589	1589
|E| (MHz)	-156	-158	-157
Lw (MHz) [H_x, H_y, H_z]	[57, 89, 65]	[77, 59, 75]	[77, 59, 75]
g	2.0033	2.0033	2.0033
Population [T_+, T_0, T_-]	[0, 1, 0]	[0, 1, 0]	[0, 1, 0]

Table S8. BPEA-Ph triplet simulation parameters, requiring differing parameters for the ST_0 / ISC triplets.

Parameters	BPEA-Ph – ZnO (3.1 nm)	BPEA-Ph – ZnO (4.1 nm)	BPEA-Ph – ZnO (6.6) nm)
|D| (MHz, ST_0 / ISC)	1600 / 1670	1590 / 1680	1590 / 1700
|E| (MHz, ST_0 / ISC)	-161 / -50	-161 / -90	-161 / -90
Lw (MHz, ST_0 / ISC) [H_x, H_y, H_z]	[77, 101, 94] / [127, 199, 200]	[37, 56, 49] / [147, 149, 150]	[37, 56, 49] / [147, 149, 150]
g	2.0033	2.0033	2.0033
ST_0 / ISC weights	0.4 / 0.6	0.6/ 0.4	0.4 / 0.6
Population [T_+, T_0, T_-] / [T_x, T_y, T_z]	[0, 1, 0] / [0.45, 0.31, 0.24]	[0, 1, 0] / [0.45, 0.39, 0.16]	[0, 1, 0] / [0.45, 0.39, 0.16]

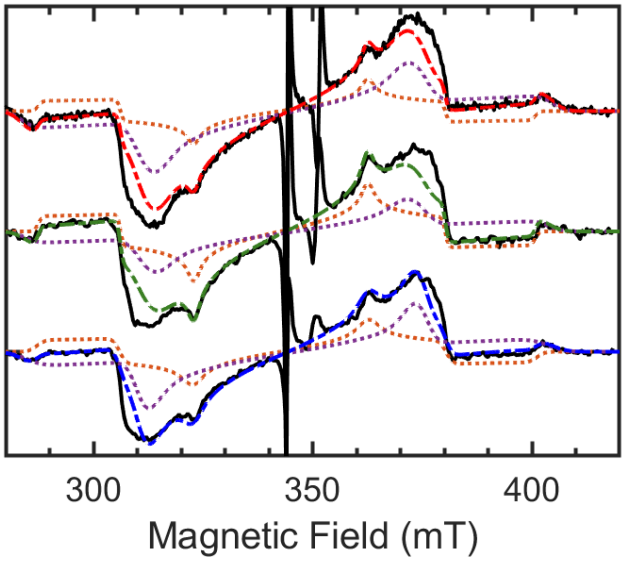

Figure S14. BPEA-Ph triplet spectra with RP-ISC (orange, dotted) and SO-ISC (purple, dotted) shown separately. Summed simulated spectra shown in blue, green, and red for 3.1, 4.1, and 6.6 nm QDs, respectively. Experimental data in black.

BPEA and BPEA-Ph Electronic Structure Calculations

All DFT calculations were performed with ORCA 5.0.4.[7,8] All calculations were performed *in vacuo* since the solvent used in the experiments (toluene) is non-polar and is not expected to have any specific interactions with the solute (BPEA or BPEA-Ph).

Radical Cation: The geometry of the molecule was optimized as radical cation (doublet state; Kohn-Sham wavefunction type UKS) using Grimme's composite electronic-structure method r^2SCAN-3c.[9] This optimized structure was used for the single point calculation/EPR parameter calculation.

EPR parameters (g-tensor, ^{1}H hyperfine tensors) were calculated for the radical cation (doublet state; Kohn-Sham wavefunction type UKS) using the B3LYP functional[10–13] in conjunction with the EPR-II basis set.[14] *g*-tensor calculations used the spin-orbit mean-field SOMF(1X) method[15] as implemented in ORCA 5.0.4.

Neutral Triplet: The geometry of the molecule was optimized as neutral triplet (Kohn-Sham wavefunction type UKS) using Grimme's composite electronic-structure method r^2SCAN-3c.[9] This optimized structure was used for the single point calculation/EPR parameter calculation.

EPR parameters (D-tensor, ^{1}H hyperfine tensors) were calculated for the neutral triplet state (Kohn-Sham wavefunction type ROKS) using the B3LYP functional[10–13] in conjunction with the EPR-II basis set.[14] Only the spin-spin part of the D-tensor was calculated. Note, that g-tensor calculations in the ROHF/ROKS framework are not implemented in this ORCA version.

BPEA – Radical Cation

Geometry Optimization: Only one imaginary frequency (<10 cm^{-1}) was found. Spin contamination was found to be negligible (≈0.007).

Single Point Calculation/EPR parameter Calculation: Spin contamination was found to be negligible (≈0.017). The spin density is substantially delocalized over the molecule which is reflected by the absence of any large ^{1}H hyperfine couplings. The small deviation of the g-values from the free electron g-value (g-tensor anisotropy ≈0.0009) is in the typical range for an organic molecule without any heavy atoms (Mulliken spin population on the two oxygen atoms is very low).

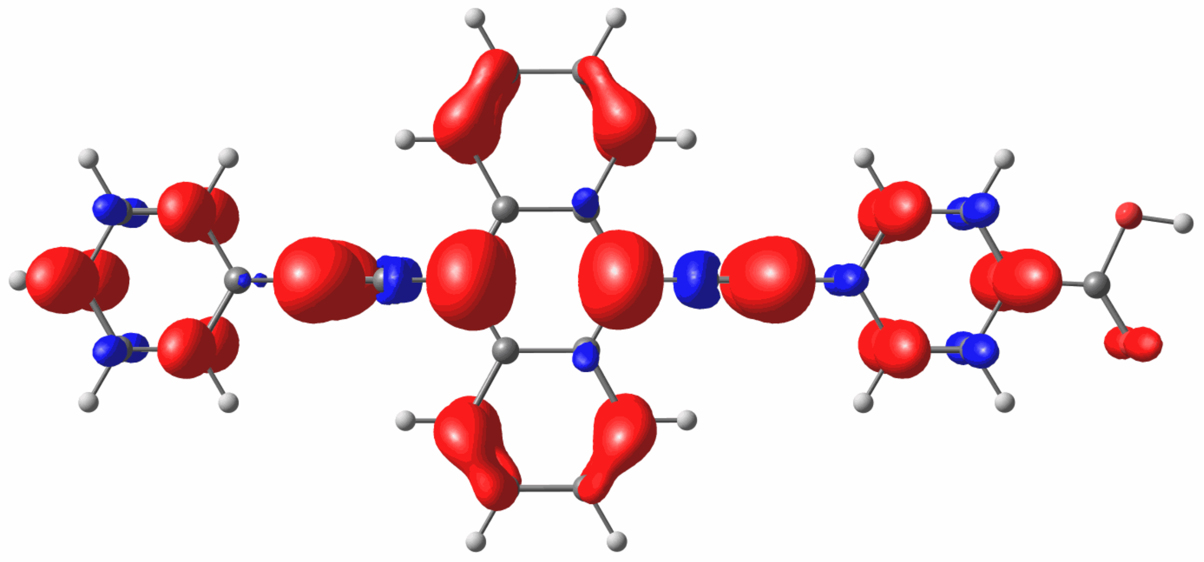

Figure S15. $BPEA^{\bullet+}$ radical cation spin density isosurface plot at the 0.002 au level.

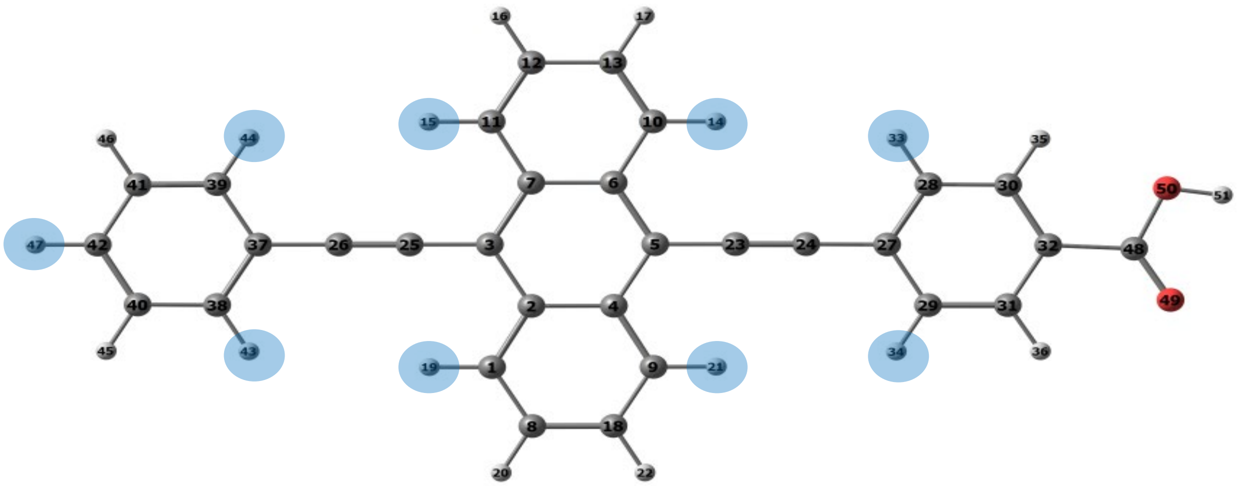

Figure S16. Protons with largest isotropic hyperfine couplings in BPEA$^{•+}$ radical cation including numbering scheme.

Table S9. $BPEA^{•+}$ radical cation g tensor and ^1H hyperfine coupling values of the protons with largest isotropic hyperfine coupling. Note, that the principal axes systems of the hyperfine tensors are differently oriented than the g-tensor principal axes system.

		$BPEA^{•+}$	g_x	g_y	g_z
		g values	2.0031	2.0029	2.0022
Nucleus	hyperfine (MHz)	A_{iso}	A_x	A_y	A_z
14H	^1H	-4.46	-5.69	-1.73	-5.97
15H	^1H	-3.89	-5.13	-1.39	-5.14
19H	^1H	-3.91	-5.15	-1.40	-5.17
21H	^1H	-4.47	-5.70	-1.73	-5.98
33H	^1H	-3.89	-4.75	-5.05	-1.87
34H	^1H	-3.75	-4.62	-4.82	-1.81
43H	^1H	-3.82	-4.72	-5.00	-1.73
44H	^1H	-3.82	-4.72	-5.00	-1.73
47H	^1H	-5.18	-5.48	-1.87	-8.19

BPEA - Neutral Triplet

Geometry Optimization: Only one imaginary frequency (11 cm^{-1}) was found. Spin contamination was found to be negligible (≈0.020).

Single Point Calculation/EPR parameter Calculation: The spin density is substantially delocalized over the molecule, but the majority is found on the central anthracene unit. The calculated ZFS parameter D is positive and about half of the experimentally determined D value. This severe underestimation of the D value in DFT calculations on polyacenes in their triplet state has been observed in previous studies.[16] (^1H hyperfine coupling values are not reported since the ROHF/ROKS calculations are not well suited for calculation of those).

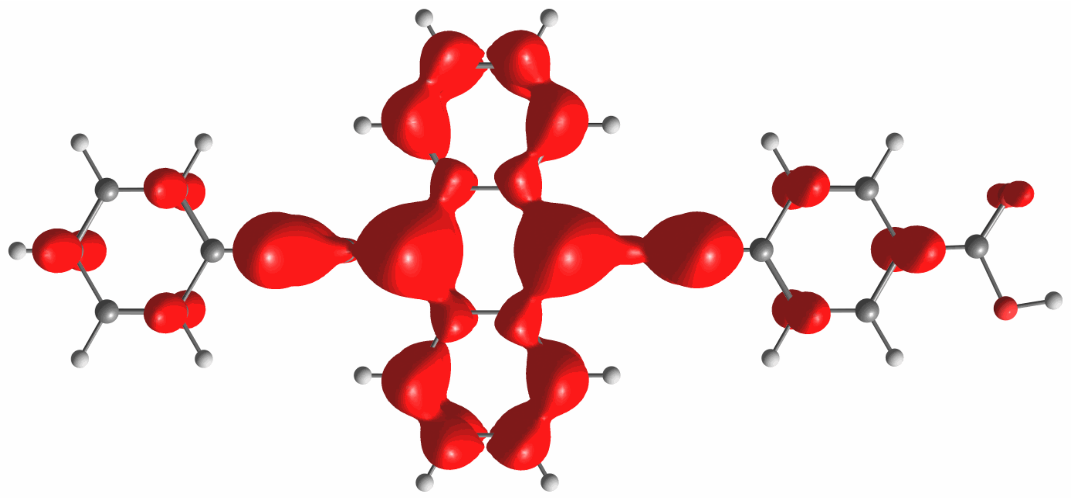

Figure S17. ^{3}BPEA neutral triplet spin density isosurface plot at the 0.002 au level.

Table S10. ^{3}BPEA neutral triplet ZFS parameters D and E.

^{3}BPEA	*D*	*E*
ZFS parameters (cm^{-1})	0.02545	0.00557
ZFS parameters (MHz)	764	167

BPEA-Ph – Radical Cation

Geometry Optimization: Only one imaginary frequency (<10 cm^{-1}) was found. Spin contamination was found to be negligible (≈0.008).

Single Point Calculation/EPR parameter Calculation: Spin contamination was found to be negligible (≈0.015). The spin density is substantially delocalized over the molecule which is reflected by the absence of any large ^1H hyperfine couplings. The small deviation of the g-values from the free electron g-value (g-tensor anisotropy ≈0.0008) is in the typical range for an organic molecule without any heavy atoms (Mulliken spin population on the two oxygen atoms is very low).

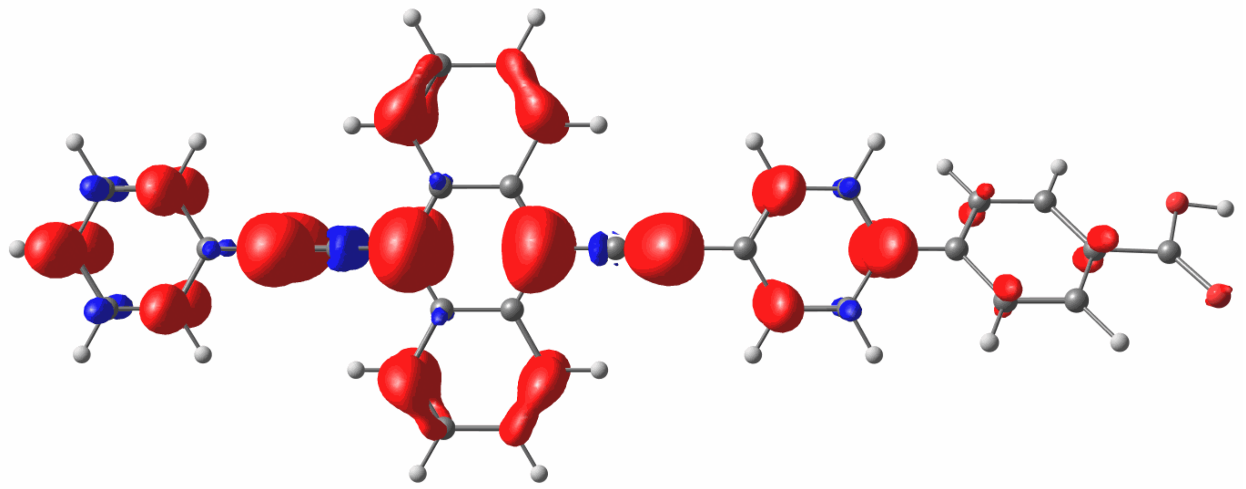

Figure S18. BPEA-Ph$^{\bullet+}$ radical cation spin density isosurface plot at the 0.002 au level.

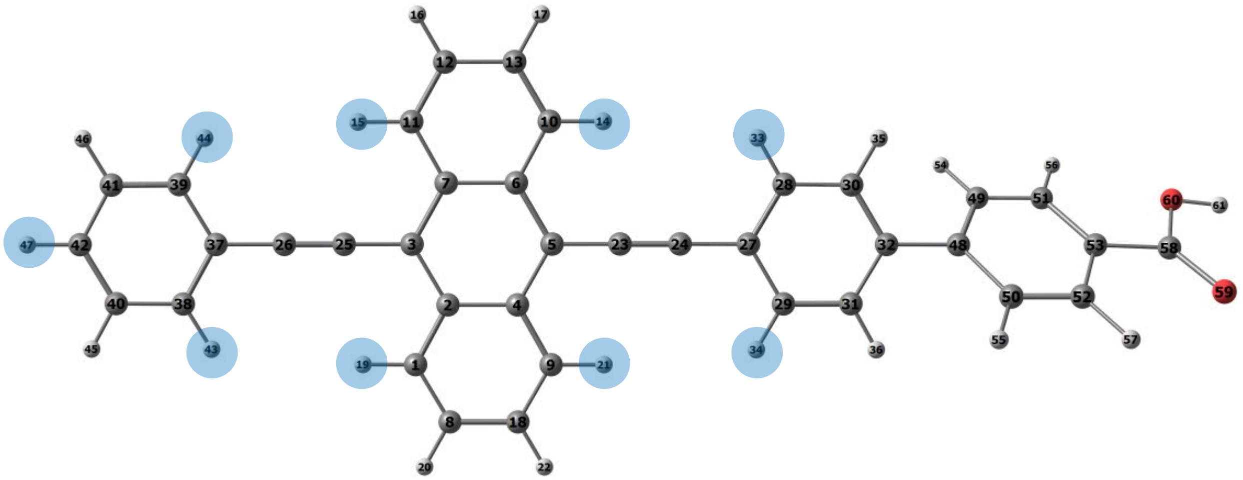

Figure S19. Protons with largest isotropic hyperfine couplings in BPEA-Ph$^{•+}$ radical cation including numbering scheme.

Table S11. BPEA-Ph$^{•+}$ radical cation *g* tensor and ^{1}H hyperfine coupling values of the protons with largest isotropic hyperfine coupling. Note, that the principal axes systems of the hyperfine tensors are differently oriented than the g-tensor principal axes system.

		BPEA-Ph$^{•+}$	g_x	g_y	g_z
		g values	2.0030	2.0029	2.0022
Nucleus	hyperfine (MHz)	A_{iso}	A_x	A_y	A_z
14H	^{1}H	-3.43	-4.62	-1.22	-4.46
15H	^{1}H	-4.00	-5.18	-1.56	-5.26
19H	^{1}H	-4.00	-5.18	-1.56	-5.27
21H	^{1}H	-3.43	-4.62	-1.22	-4.46
33H	^{1}H	-3.53	-4.42	-4.64	-1.53
34H	^{1}H	-3.53	-4.42	-4.64	-1.53
43H	^{1}H	-3.72	-4.59	-4.85	-1.72
44H	^{1}H	-3.72	-4.59	-4.85	-1.72
47H	^{1}H	-4.91	-5.18	-1.78	-7.78

BPEA-Ph – Neutral Triplet

Geometry Optimization: Only one imaginary frequency (<11 cm^{-1}) was found. Spin contamination was found to be negligible (≈0.02).

Single Point Calculation/EPR parameter Calculation: The spin density is substantially delocalized over the molecule, but the majority is found on the central anthracene unit. The calculated ZFS parameter D is positive and about half of the experimentally determined D value. ZFS parameters (Table S12) are very similar to those calculated for ^{3}BPEA (Table S10), which is expected since hardly any spin density is found on the additional phenyl group in BPEA-Ph. The severe underestimation of the D value in DFT calculations on polyacenes in their triplet state has been observed in previous studies.[16] (^{1}H hyperfine coupling values are not reported since the ROHF/ROKS calculations are not well suited for calculation of those).

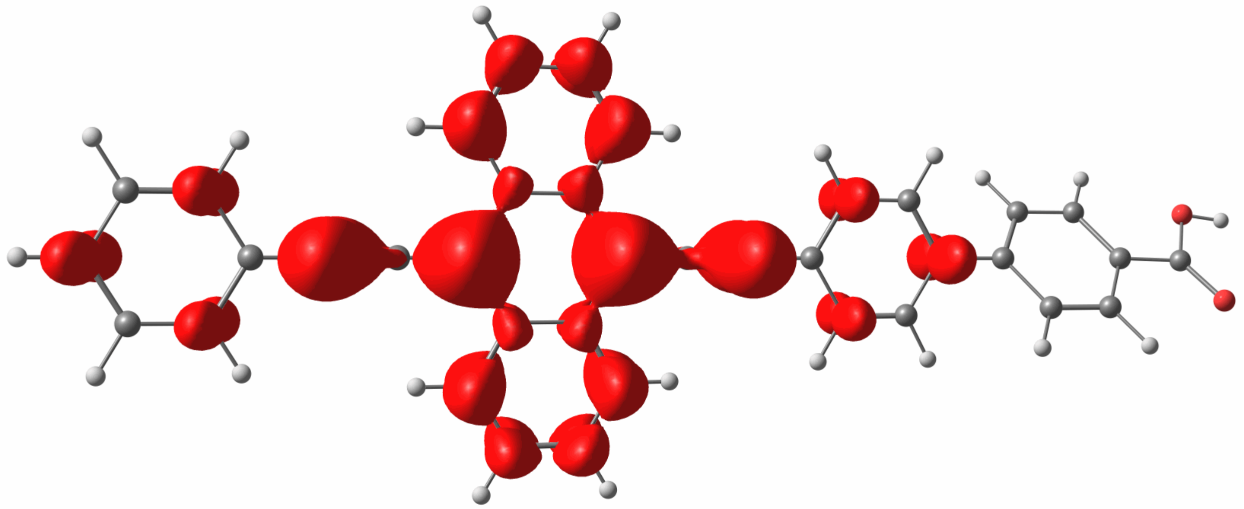

Figure S20. ^{3}BPEA-Ph neutral triplet spin density isosurface plot at the 0.002 au level.

Table S12. ^{3}BPEA neutral triplet ZFS parameters D and E.

^{3}BPEA-Ph	*D*	*E*
ZFS parameters (cm⁻¹)	0.02553	0.00547
ZFS parameters (MHz)	766	164

References

(1) Rossini, J. E.; Huss, A. S.; Bohnsack, J. N.; Blank, D. A.; Mann, K. R.; Gladfelter, W. L. Binding and Static Quenching Behavior of a Terthiophene Carboxylate on Monodispersed Zinc Oxide Nanocrystals. *J. Phys. Chem. C* **2011**, *115* (1), 11–17. https://doi.org/10.1021/jp1080143.

(2) N. Oehrlein, A.; Sanchez-Diaz, A.; C. Goff, P.; M. Ziegler, G.; M. Pappenfus, T.; R. Mann, K.; A. Blank, D.; L. Gladfelter, W. Effects of a Phosphonate Anchoring Group on the Excited State Electron Transfer Rates from a Terthiophene Chromophore to a ZnO Nanocrystal. *Phys. Chem. Chem. Phys.* **2017**, *19* (35), 24294–24303. https://doi.org/10.1039/C7CP03784H.

(3) Oehrlein, A. N.; Sanchez-Diaz, A.; Goff, P. C.; Planells, M.; Robertson, N.; Blank, D. A.; Gladfelter, W. L. Effect of Extending Conjugation via Thiophene-Based Oligomers on the Excited State Electron Transfer Rates to ZnO Nanocrystals. *Phys. Chem. Chem. Phys.* **2019**, *21* (13), 6991–6998. https://doi.org/10.1039/C9CP00420C.

(4) Hoyer, P.; Weller, H. Size-Dependent Redox Potentials of Quantized Zinc Oxide Measured with an Optically Transparent Thin Layer Electrode. *Chem. Phys. Lett.* **1994**, *221*, 379–384.

(5) Ringström, R.; Schroeder, Z. W.; Mencaroni, L.; Chabera, P.; Tykwinski, R. R.; Albinsson, B. Triplet Formation in a 9,10-Bis(Phenylethynyl)Anthracene Dimer and Trimer Occurs by Charge Recombination Rather than Singlet Fission. *J. Phys. Chem. Lett.* **2023**, *14* (35), 7897–7902. https://doi.org/10.1021/acs.jpclett.3c02050.

(6) Matsui, M.; Kotani, M.; Kubota, Y.; Funabiki, K.; Jin, J.; Yoshida, T.; Higashijima, S.; Miura, H. Comparison of Performance between Benzoindoline and Indoline Dyes in Zinc Oxide Dye-Sensitized Solar Cell. *Dyes Pigments* **2011**, *91* (2), 145–152. https://doi.org/10.1016/j.dyepig.2011.02.009.

(7) Neese, F.; Wennmohs, F.; Becker, U.; Riplinger, C. The ORCA Quantum Chemistry Program Package. *J. Chem. Phys.* **2020**, *152* (22), 224108. https://doi.org/10.1063/5.0004608.

(8) Neese, F. Software Update: The ORCA Program System—Version 5.0. *WIREs Comput. Mol. Sci.* **2022**, *12* (5), e1606. https://doi.org/10.1002/wcms.1606.

(9) Grimme, S.; Hansen, A.; Ehlert, S.; Mewes, J.-M. r2SCAN-3c: A "Swiss Army Knife" Composite Electronic-Structure Method. *J. Chem. Phys.* **2021**, *154* (6), 064103. https://doi.org/10.1063/5.0040021.

(10) Becke, A. D. Density-functional Thermochemistry. I. The Effect of the Exchange-only Gradient Correction. *J. Chem. Phys.* **1992**, *96* (3), 2155–2160. https://doi.org/10.1063/1.462066.

(11) Lee, C.; Yang, W.; Parr, R. G. Development of the Colle-Salvetti Correlation-Energy Formula into a Functional of the Electron Density. *Phys. Rev. B* **1988**, *37* (2), 785–789. https://doi.org/10.1103/PhysRevB.37.785.

(12) Vosko, S. H.; Wilk, L.; Nusair, M. Accurate Spin-Dependent Electron Liquid Correlation Energies for Local Spin Density Calculations: A Critical Analysis. *Can. J. Phys.* **1980**, *58* (8), 1200–1211. https://doi.org/10.1139/p80-159.

(13) Stephens, P. J.; Devlin, F. J.; Chabalowski, C. F.; Frisch, M. J. Ab Initio Calculation of Vibrational Absorption and Circular Dichroism Spectra Using Density Functional Force Fields. *J. Phys. Chem.* **1994**, *98* (45), 11623–11627. https://doi.org/10.1021/j100096a001.

(14) Barone, V. Structure, Magnetic Properties and Reactivities of Open-Shell Species From Density Functional and Self-Consistent Hybrid Methods. In *Recent Advances in Density*

Functional Methods; Recent Advances in Computational Chemistry; WORLD SCIENTIFIC, 1995; Vol. Volume 1, pp 287–334. https://doi.org/10.1142/9789812830586_0008.
(15) Neese, F. Efficient and Accurate Approximations to the Molecular Spin-Orbit Coupling Operator and Their Use in Molecular g-Tensor Calculations. *J. Chem. Phys.* **2005**, *122* (3), 034107. https://doi.org/10.1063/1.1829047.
(16) Sinnecker, S.; Neese, F. Spin−Spin Contributions to the Zero-Field Splitting Tensor in Organic Triplets, Carbenes and BiradicalsA Density Functional and Ab Initio Study. *J. Phys. Chem. A* **2006**, *110* (44), 12267–12275. https://doi.org/10.1021/jp0643303.
